# Patient Health Questionnaire-9 Item Pairing Predictiveness for Prescreening Depressive Symptomatology: Machine Learning Analysis

**DOI:** 10.2196/48444

**Published:** 2023-10-19

**Authors:** Darragh Glavin, Eoin Martino Grua, Carina Akemi Nakamura, Marcia Scazufca, Edinilza Ribeiro dos Santos, Gloria H Y Wong, William Hollingworth, Tim J Peters, Ricardo Araya, Pepijn Van de Ven

**Affiliations:** 1 Department of Electronic and Computer Engineering University of Limerick Limerick Ireland; 2 Health Research Institute University of Limerick Limerick Ireland; 3 Departamento de Psiquiatria Faculdade de Medicina Universidade de Sao Paulo Sao Paulo Brazil; 4 Instituto de Psiquiatria, Hospital das Clinicas Faculdade de Medicina Universidade de Sao Paulo Sao Paulo Brazil; 5 Health Sciences School University of the State of Amazonas Manaus Brazil; 6 Department of Social Work and Social Administration University of Hong Kong Pok Fu Lam China (Hong Kong); 7 Sau Po Centre on Ageing University of Hong Kong Pok Fu Lam China (Hong Kong); 8 Bristol Medical School University of Bristol Bristol United Kingdom; 9 Bristol Dental School University of Bristol Bristol United Kingdom; 10 Centre for Global Mental Health Institute of Psychiatry King's College London London United Kingdom

**Keywords:** Patient Health Questionnaire-2, PHQ-2, Patient Health Questionnaire-9, PHQ-9 items, depressive symptomatology, ultrabrief questionnaires, prescreening, machine learning, cardinal symptoms, low energy, psychomotor dysfunction, depressed mood

## Abstract

**Background:**

*Anhedonia* and *depressed mood* are considered the cardinal symptoms of major depressive disorder. These are the first 2 items of the Patient Health Questionnaire (PHQ)–9 and comprise the ultrabrief PHQ-2 used for prescreening depressive symptomatology. The prescreening performance of alternative PHQ-9 item pairings is rarely compared with that of the PHQ-2.

**Objective:**

This study aims to use machine learning (ML) with the PHQ-9 items to identify and validate the most predictive 2-item depressive symptomatology ultrabrief questionnaire and to test the generalizability of the best pairings found on the primary data set, with 6 external data sets from different populations to validate their use as prescreening instruments.

**Methods:**

All 36 possible PHQ-9 item pairings (each yielding scores of 0-6) were investigated using ML-based methods with logistic regression models. Their performances were evaluated based on the classification of depressive symptomatology, defined as PHQ-9 scores ≥10. This gave each pairing an equal opportunity and avoided any bias in item pairing selection.

**Results:**

The ML-based PHQ-9 items 2 and 4 (phq2&4), the *depressed mood* and *low-energy* item pairing, and PHQ-9 items 2 and 8 (phq2&8), the *depressed mood* and *psychomotor retardation or agitation* item pairing, were found to be the best on the primary data set training split. They generalized well on the primary data set test split with area under the curves (AUCs) of 0.954 and 0.946, respectively, compared with an AUC of 0.942 for the PHQ-2. The phq2&4 had a higher AUC than the PHQ-2 on all 6 external data sets, and the phq2&8 had a higher AUC than the PHQ-2 on 3 data sets. The phq2&4 had the highest Youden index (an unweighted average of sensitivity and specificity) on 2 external data sets, and the phq2&8 had the highest Youden index on another 2. The PHQ-2≥2 cutoff also had the highest Youden index on 2 external data sets, joint highest with the phq2&4 on 1, but its performance fluctuated the most. The PHQ-2≥3 cutoff had the highest Youden index on 1 external data set. The sensitivity and specificity achieved by the phq2&4 and phq2&8 were more evenly balanced than the PHQ-2≥2 and ≥3 cutoffs.

**Conclusions:**

The PHQ-2 did not prove to be a more effective prescreening instrument when compared with other PHQ-9 item pairings. Evaluating all item pairings showed that, compared with alternative partner items, the *anhedonia* item underperformed alongside the *depressed mood* item. This suggests that the inclusion of *anhedonia* as a core symptom of depression and its presence in ultrabrief questionnaires may be incompatible with the empirical evidence. The use of the PHQ-2 to prescreen for depressive symptomatology could result in a greater number of misclassifications than alternative item pairings.

## Introduction

### Background

The Diagnostic and Statistical Manual of Mental Disorders, fifth edition (DSM-V) defines *anhedonia* (inability to find pleasure in what would usually be pleasurable activities) and *depressed mood* as the core criteria of major depressive disorder (MDD) [1]. According to the DSM-V, at least 1 of these cardinal symptoms must be present for a period of at least the past 2 weeks for a positive diagnosis of MDD, along with ≥5 symptoms. The heightened importance of these symptoms is also seen in screening questionnaires derived from the DSM-V’s diagnostic criteria for MDD such as the depression module of the Primary Care Evaluation of Mental Disorders [2]; the depression module of the Patient Health Questionnaire (PHQ); the PHQ-9 [3]; and its ultrabrief version, PHQ-2 [4]. This importance stems from a consensus-based approach to the diagnosis of MDD formed from clinical experience [5].

The PHQ-9 has gained widespread popularity since its introduction and is now the most reliable and commonly used screening instrument in primary care and clinical research [6,7]. Each item of the PHQ-9 corresponds to a symptom of MDD: anhedonia, depressed mood, sleep disturbances, fatigue, appetite changes, feelings of worthlessness and excessive guilt, concentration difficulties, psychomotor disturbances, and suicidal ideation. The PHQ-9 assesses symptom frequency over the previous 2 weeks. This is more in line with the criteria required for an MDD diagnosis according to the DSM-V than the Primary Care Evaluation of Mental Disorders’ 1-month assessment. Responses for the items are 0 (*Not at all*), 1 (*Several days*), 2 (*More than half the days*), and 3 (*Nearly every day*). The PHQ-9 was originally intended for use as a diagnostic algorithm and a severity-level measure. The PHQ-9 algorithm requires a minimum of 5 items to be scored as ≥2, with at least 1 of the first 2 items: (1) *a loss of interest or pleasure in doing things* and (2) *feeling down, depressed, or hopeless* endorsed as part of the 5 items. Although the algorithm structure closely matches the MDD criterion of the DSM-V, simply applying a ≥10 cutoff to the PHQ-9 severity scores has proven to be a reliable screening instrument for depression with performance equal to or better than the algorithm [3,6,7].

The PHQ-2 contains only the 2 core MDD symptom criteria, *anhedonia* (PHQ-9 item 1) and a *depressed mood* (PHQ-9 item 2 [phq2]). Its performance as a prescreening instrument for depressive symptomatology has been validated against other longer questionnaires [8]. However, in a diagnostic meta-analysis of 21 studies, the PHQ-2 has been shown to have lower accuracy in identifying MDD than initially reported [9]. The predictiveness of individual MDD symptoms has been previously compared [10]. However, symptoms are rarely evaluated when combined as pairings, and when they are, no pairing shows standout superiority [11]. As previously mentioned, the 2 items in the PHQ-2 were chosen as they are deemed the cardinal symptoms of depression, but objective evidence that these are indeed the 2 best items in a 2-item PHQ is lacking. Depressive symptomatology cases may go undetected if the cardinal symptoms are a suboptimal item pairing. Moreover, applying a cutoff to the summation of responses is a rather arbitrary approach, which is likely mainly used to date because of its simplicity. Machine learning (ML) offers a logical approach to tackle both issues. ML algorithms allow a structured, data-driven approach to item selection, thus allowing the selection of the most predictive 2 questions of the PHQ-9. ML algorithms are not limited to the restrictive summation and greater-than-or-equal-to logic used in the PHQ-2 but rather allow for the weighing of individual items as well as nonlinear transformations of the sum score. Consequently, this results in more thresholds to fine-tune instrument performance, leading to more refined classifications of depressive symptomatology.

### Prior Work

To our knowledge, our previous analysis is the only one to date that has compared the performance of the PHQ-2 with alternative PHQ-9 item pairings and explored their combination with ML algorithms to predict depressive symptomatology [12]. ML algorithms have previously been combined with PHQ-9 items, but it was used to accurately predict suicidal ideation [13]. Our earlier work provided a data-driven ML analysis of all 36 possible PHQ-9 item pairings to predict depressive symptomatology [12]. Depressive symptomatology was defined as PHQ-9 scores ≥10 and was the reference standard used. This data-driven ML approach investigated the underlying relationship between symptoms and depressive symptomatology, without imposing any preconceptions on symptom importance.

Random oversampling of the screen positive class (ie, PHQ-9 scores ≥10) balanced the output classes to assist ML models in accurately predicting this less-frequent class. For direct comparison, the classification performance of all pairings was compared with the PHQ-2 on the same samples. The item pairings of PHQ-9 items 2 and 4 (phq2&4), the *depressed mood* and low*-*energy symptoms, and PHQ-9 items 2 and 8 (phq2&8), the *depressed mood* and *psychomotor retardation or agitation* symptoms, achieved the highest area under the receiver operating characteristic (ROC) curves out of all possible PHQ-9 item pairings, including the PHQ-2 on both cross-validation (CV) and test data. This strong generalization performance achieved by the phq2&4 and phq2&8 on the out-of-sample test data indicated the potential use of these new pairings as prescreening instruments.

### Objectives

The first objective was to re-evaluate the ML-based phq2&4 and phq2&8 on the primary data set with oversampling removed. Our previous analysis [12] used random oversampling to balance the output classes (PHQ-9<10 and PHQ-9≥10) and prevent the more frequent class from inflating performance scores, such as accuracy. However, as ROC curve analysis and area under the curve (AUC) are irrespective of class balance, there was no need to oversample; therefore, the analysis of the primary data set was rerun in this study. Next, the main objective of this work was to validate the new phq2&4 and phq2&8 pairings by investigating their generalization performance on 6 external data sets that were not used during the training of the ML models. These external data sets provided new samples with various demographics to test the pairing ability on out-of-sample data.

The length of the ultrabrief questionnaire was limited to 2 items because the performance of the PHQ-2, considered the gold standard ultrabrief version of the PHQ-9, served as the baseline. Maintaining a constant questionnaire length enabled the evaluation of alternative pairings compared with the established PHQ-2. To validate their ability as prescreening instruments, the phq2&4 and phq2&8 must generalize well and outperform the PHQ-2 on these external data sets. The fixed length also facilitated a comparison between a new ML methodology for screening and the traditional sum score psychometric approach used with the PHQ-2. Pairing performance was only evaluated for adult populations, aged ≥18 years, as an alternative questionnaire to the PHQ-9, the PHQ-Adolescents [14], exists for identifying depressive symptomatology in adolescents.

## Methods

### Data Sources

#### Overview

This analysis used 7 data sets (Table 1). The ML models were trained on the primary data set training split, and the best pairings were selected based on their CV performance on this split. The generalization performance of the best pairings was first estimated using a test split of the primary data set. To analyze this further, outside of this test split, 6 external data sets with various participant demographics were sourced. Four of the external data sets represented a wider Brazilian population (Pesquisa Nacional de Saúde 2013 [PNS2013], Pesquisa Nacional de Saúde 2019 [PNS2019], Amazonas, and São Paulo-Manaus). The other 2 represented different populations (Mexican Medical Students [MexMedStudents] and Jockey Club [JC] JoyAge). Two of the external data sets represented age demographics similar to the primary data set (São Paulo-Manaus and JC JoyAge). The performance on these 6 external data sets determined if the best pairings generalized well to wider adult populations and would be viable prescreening instruments with performance equal to or better than the PHQ-2.

**Table 1:** Overview of the characteristics of the 7 data sets presented.

Data set	Country	Year	Sample size, n	Age (y), mean (SD; range)	Sex (female), n (%)	PHQ-9^a^ scores, mean (SD)	PHQ-9 scores ≥10, n (%)
PROACTIVE	Brazil	2019	4025	68.4 (6.5; 60-100)	2542 (63.2)	6.9 (7.0)	1216 (30.2)
PNS2013^b^	Brazil	2013	60,202	43.3 (16.7; 18-101)	34,282 (56.9)	2.8 (4.3)	5051 (8.4)
PNS2019^c^	Brazil	2019	88,531	47.2 (17.1; 18-107)	46,869 (52.9)	3.4 (4.7)	9252 (10.5)
Amazonas	Brazil	2013-2014	1631	40.2 (15.4; 20-94)	838 (51.4)	5.3 (5.4)	313 (19.2)
São Paulo-Manaus	Brazil	2010-2011	1377	72.5 (8.5; 60-104)	759 (55.1)	3.2 (4.1)	117 (8.5)
Mexican Medical Students	Mexico	2014	772	20.2 (1.8; 18-31)	399 (51.7)	7.2 (4.4)	192 (24.9)
Jockey Club JoyAge	Hong Kong	2018-2019	4221	77.3 (8.8; 60-118)	3274 (77.6)	6.7 (4.2)	809 (19.2)

^a^PHQ-9: Patient Health Questionnaire–9.

^b^PNS2013: Pesquisa Nacional de Saúde 2013.

^c^PNS2019: Pesquisa Nacional de Saúde 2019.

The data sets were preprocessed independently of one another. PHQ-9 item responses were either missing or within the expected 0-3 Likert scale range. Samples with >2 missing item responses were removed from the data sets. Any remaining missing responses were imputed with the respective item’s mode because of the ordinal nature of the item responses.

#### Primary Data Set

The PROACTIVE study was a cluster randomized controlled trial conducted in socioeconomically deprived areas of Guarulhos, Brazil, where a psychosocial intervention was provided to older adults that aimed to reduce depressive symptoms [15,16]. Individuals registered with primary care clinics were randomly interviewed for recruitment into the randomized controlled trial and were screened for depressive symptomatology using the PHQ-9 in a dedicated application on an Android tablet (n=4034) [17]. The primary data set used for this analysis was the PROACTIVE screening data set, which was used to train, cross-validate, and initially test all ML models for different PHQ-9 item pairings. Nine individuals did not complete the PHQ-9 questionnaire and were excluded (n=4025). PHQ-9 scores ≥10 were used to classify an individual as having depressive symptomatology and was the principal inclusion criterion for the trial. The data set split was 69.99% (2817/4025) for training and 30.01% (1208/4025) for testing. The training set was further split into 5 folds for CV (approximately n=563 each). In a single CV iteration, 4 of these folds were used to train the ML models and the fifth fold was used for validation. All 5 CV iterations used a different validation fold and subsequently 4 different training folds, until all 5 folds were used for validation. The average performance across the 5 validation folds provided an initial out-of-sample performance estimate.

#### External Data Sets

##### Brazilian National Health Survey (Pesquisa Nacional de Saúde)

The PHQ-9 was self-administered in 2 Brazilian national health surveys: one in 2013, PNS2013 (n=222,385), and another in 2019, PNS2019 (n=293,726) [18]. Both data sets provided an evaluation of the pairing generalizability on a broader Brazilian population and outside of a primary care setting. The screening interviews for the primary data set were also conducted in 2019, removing any potential temporal effect on the generalization performance between it and this PNS2019 data set. As this analysis focused on the generalization performance of pairings in adult populations, only PHQ-9 responses from those aged ≥18 years were used (PNS2013: n=145,580 and PNS2019: n=207,845). Individuals who responded to <7 of the PHQ-9 items were excluded (PNS2013: n=60,202 and PNS2019: n=88,531).

##### Amazonas

Adults registered with primary care clinics in Coari (n=805) and Tefe (n=826), 2 cities in the State of Amazon, Brazil, completed the PHQ-9 during interviews for a cross-sectional study of depressive symptomatology prevalence, defined as PHQ-9 scores ≥10, and depression care [19]. Individuals aged ≥20 years were randomly selected from a database of eligible participants (n=1631). Interviews were held at individuals’ homes upon consenting to participation and were conducted between August 2013 and May 2014.

##### São Paulo-Manaus

This data set comprised PHQ-9 responses from older adults registered with primary care clinics and primary health care professionals (n=1380) in São Paulo (n=703) and Manaus (n=677), Brazil. The study aimed to investigate the public stigma surrounding depression in older adults. The PHQ-9 was administered via interview to assess depressive symptoms in this sample and how these affected stigmatization [20]. Older adults were defined as those aged ≥60 years, the same age demographic as the primary data set, which provided a constant factor within the generalization analysis of this data set. São Paulo city and Guarulhos are both in São Paulo state, another constant factor for a portion of this sample, and Manaus is from the Amazon state, similar to the Amazonas data set. Participants were randomly selected, stratified by age and sex. Three participants with no PHQ-9 responses were excluded (n=1377).

##### Mexican Medical Students

Medical students registered at a private Mexican university were randomly selected to self-administer a mental health survey, which contained the PHQ-9 among other questionnaires (n=1200) [21]. Of those selected, about two-thirds consented to participate; of these, 2 students aged <18 years were excluded from the analysis because it is concerned with generalization performance in adults, along with 2 adults without PHQ-9 responses (n=772).

##### JC JoyAge

Jockey Club Holistic Support Project for Elderly Mental Wellness (JC JoyAge) is a community-based mental health service for older adults in Hong Kong [22]. Adults aged ≥60 years completed the PHQ-9 to evaluate depressive symptoms during an assessment interview after referral to the project by peer supporters in the community (n=4267). A total of 46 individuals were excluded owing to missing >2 PHQ-9 item responses (n=4221). There was a higher proportion of mild depressive symptoms (PHQ-9 scores from 5 to 9) in this sample, potentially because of the referral aspect of the study.

### Instruments

#### PHQ-2 Instrument

The PHQ-2 [4] is an ultrabrief questionnaire that contains the first 2 items of the PHQ-9: *little interest or pleasure in doing things* and *feeling down,* *depressed, or hopeless*. Responses are on the same scale as the parent PHQ-9, “Not at all” to “Nearly every day,” but the total scores range from 0 to 6. The optimal PHQ-2 score cutoff for classifying depressive symptomatology has been debated. The original study suggested a cutoff of ≥3 for optimal performance [4]. This was validated as the optimal threshold in other studies [7,8], but some studies required a lower cutoff of ≥2 to maximize sensitivity and specificity [9].

#### ML-Based Pairings

In our previous analysis, the phq2&4 and phq2&8 were found to best classify individuals into screen positive and screen negative cases of depressive symptomatology [12]. The phq2&4 contains the second and fourth items of the PHQ-9, and the phq2&8 contains the second and eighth items. As both contain 2 PHQ-9 items, their total scores also range from 0 to 6. However, these ML-based instruments do not use greater-than-or-equal-to cutoffs to classify individuals in the screen negative and screen positive groups. Instead, they use some (generally nonlinear) function applied to the inputs to calculate an output. In the case of logistic regression (LR), this output is a linear combination of the inputs, which is then nonlinearly transformed to an output domain of 0 to 1 by the sigmoid function. This output can be interpreted as a probability score for class membership of the presented input. The classification performance of the model can be adjusted by tuning the probability threshold for which a certain input is deemed to belong to 1 of the classes. As the 2 items that formed the input pairings for these models can each take on 4 values, the input space of these models consisted of 4^2^=16 different patterns. These 16 patterns in turn led to 16 different probability thresholds that could be chosen to obtain a desired model performance.

### ML Analysis

The ML methodology applied to the primary data set in this study was based on the work done in our previous analysis [12]. As in the previous analysis, all 36 unique PHQ-9 item pairings were iterated. Each pairing trained an ML model for a complete performance comparison between all pairings. Random oversampling of the less-frequent screen positive class was removed from this analysis. Random oversampling is typically performed in ML analyses to balance the output classes to prevent strong performance on the more frequent class inflating the accuracy. As the accuracy of the item pairings was not the main metric evaluated in this analysis, there was no need to duplicate samples of the less-frequent class to balance the classes. Instead, ROC curve analysis and AUC, which are irrespective of class balance, investigated pairing performance for multiple thresholds. The primary data set was split into a training and a test set. A 5-fold CV was applied to the training set to obtain initial out-of-sample performance insights during the training process, without exposing the test set. Multiple ML algorithms were evaluated to optimize the classification of depressive symptomatology. The LR, decision tree, extreme gradient boosting, support vector classifier, and multilayer perceptron ML algorithms were evaluated.

All ML algorithms, except for decision trees achieved similar CV performance. To simplify the pipeline, LR models were chosen because (1) these are well-established models in both the statistics and ML domains, (2) ease of training, and (3) interpretability of model coefficients. To make classifications using an LR model, the probability of a given set of inputs (a pairing response combination) belonging to the positive class (PHQ-9≥10) is estimated. The inputs are linearly combined using the following multiple linear regression equation:

*z = w_1_.phq_i_ + w_2_.phq_j_ + b* **(1)**,

where *w_1_* and *w_2_* represent the weights that multiply the 2 PHQ items *phq_i_* and *phq_j_*, and *b* represents the bias term. Subsequently, the resulting sum of this equation (*z*) is nonlinearly transformed using the sigmoid function:

*ŷ = 1 / 1+e^-z^* **(2)**,

which bounds the output (*ŷ*) to a value between 0 and 1. This output can be interpreted as a probability score for class membership for a given set of inputs. If the output probability is above a set threshold (≥0.5 is the default), a screen positive prediction is made.

Separate LR models were trained for each item pairing on the training data set. The optimal LR regularization hyperparameter value was selected for each model based on the CV AUC performance through Bayesian hyperparameter tuning [23]. Each LR model’s weights and bias were learned from the data during the training process. The models learn the association between the 2 item responses and PHQ-9 scores ≥10 and how to best classify individuals as screen positive or screen negative. The weights and bias were updated throughout the training process, and the CV performance was evaluated by comparing the predictions with the real outputs. The phq2&4 and phq2&8 multiple linear regression equations and hyperparameters are provided in Multimedia Appendix 1. By withholding a proportion of the data for validation during CV, the model generalization on unseen data can be estimated. Without performing CV, models may overfit on the training data, that is, they may learn the specifics of the training data too well and may generalize poorly on out-of-sample data.

The performance of both the ≥2 and ≥3 cutoffs was reported for the PHQ-2, as these are the most commonly reported [9]. For the ML models, the threshold was chosen from the 16 candidates to maximize the Youden index (sensitivity + specificity − 1) based on CV performance on the training split of the primary data set. The maximized Youden index is a common selection criterion for an optimal threshold, as it weighs sensitivity and specificity equally [24]. Although it may not correspond to the optimal threshold in all prescreening contexts, it avoids any personal biases on the importance of sensitivity or specificity influencing the reported results.

Pairings were ranked based on their mean AUC score for the ROC across the 5 CV folds. The AUC scores measure the overall performance of a binary classifier irrespective of the chosen threshold. The predictive ability of the 2 best-performing PHQ-9 item pairings was compared with that of the PHQ-2. As the PHQ-2 does not need to be trained (ie, learn how best to classify individuals as screen negative or screen positive), its CV scores were calculated by manually assessing its performance on the same 5 CV folds on which the ML models were evaluated. The generalization performance of the best pairings and their optimal thresholds were then evaluated on the primary data set’s test set and the 6 external data sets. These external data sets were used solely for testing, meaning the pairing ML models were not retrained and so did not learn new information from these external data sets. Their purpose was solely to investigate the performance of the new pairings as depressive symptomatology prescreening instruments and to compare it with that of the PHQ-2. The thresholds were not adjusted to suit the external data, and therefore, any data-driven optimal threshold reporting bias was removed [24].

Other metrics, such as positive predictive value (PPV) and negative predictive value (NPV), were reported, but the thresholds were not optimized for these. A complete performance report for all thresholds is provided in Multimedia Appendix 2. All the data preprocessing and ML models were coded using *Python 3.9* (Python Software Foundation). *Pandas 1.4.4* was used for data management. The entire ML pipeline was constructed using *Scikit-Learn 1.1.1*. The *BayesSearchCV* function in *Scikit-optimize 0.9.0* provided the algorithm evaluation and hyperparameter optimization. Graphs were plotted using *Matplotlib 3.5.2*.

### Ethical Considerations

The PROACTIVE trial obtained written informed consent from participants before face-to-face interviews, and verbal consent was obtained for telephone interviews. The trial was approved by the Research Ethics Committee of the University of São Paulo Medical School (Comitê de Ética em Pesquisa Faculdade de Medicina da Universidade de São Paulo 2.836.569) and was authorized by the Guarulhos Health Secretary. The Brazilian National Health Ethics Research Committee of the Brazilian National Health Council approved both the PNS2013 and PNS2019 surveys. All the participants signed an informed consent form. Anonymized versions of both the PNS2013 and PNS2019 surveys are publicly available for download and analysis. The University of São Paulo Medical School Ethical Committee approved the Amazonas study. The Health Secretariat of the municipalities of Coari and Tefe consented to the study, and written informed consent was obtained from the participants.

The São Paulo-Manaus study procedures were approved by the Research Ethics Committee of the Faculty of Medicine at the University of São Paulo, the Municipal Secretary of Health of São Paulo, the University of Amazonas State, and the Municipal Secretary of Health of Manaus. The participants provided written informed consent before data collection began. The MexMedStudents study complied with the ethical considerations stipulated in the Helsinki Treaties, Good Clinical Practices, and Ethics and Epidemiology: International Guidelines. The participants provided written informed consent for voluntary participation in the study and the use of their data. This data set is publicly available on the web. The JC JoyAge study received ethics approval from the Human Research Ethics Committee of the University of Hong Kong (reference EA1709021). All the participants provided informed consent.

None of the data sets used in this analysis contained personally identifiable information. None of the participants received financial compensation for their participation in their respective studies.

## Results

### Primary Data Set

#### Probability Thresholds

**Figure 1:**
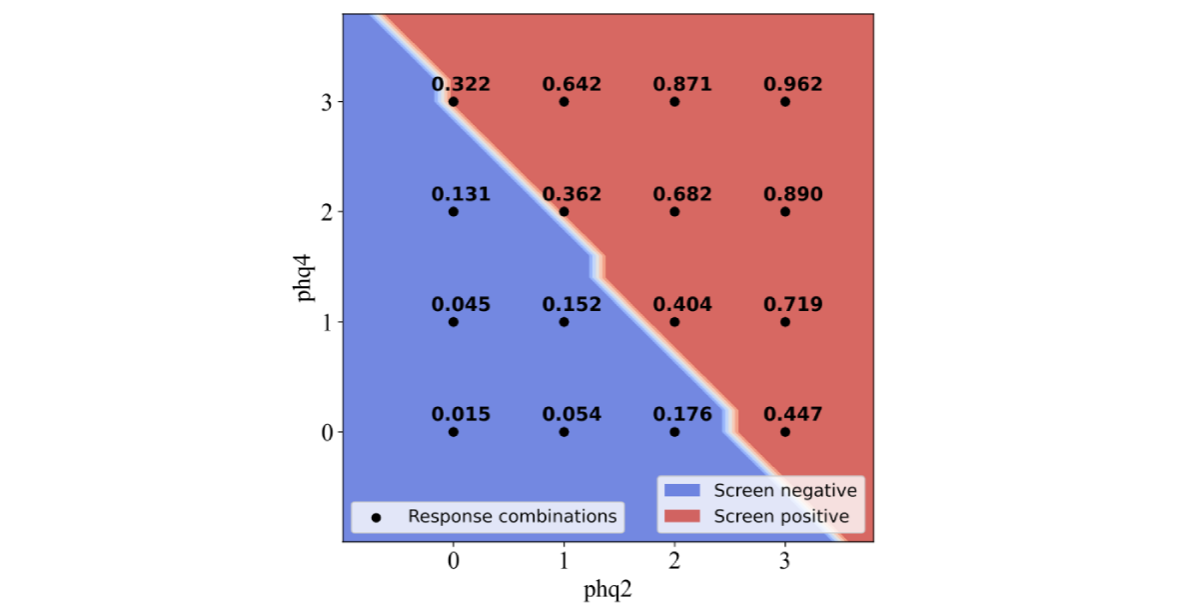
Input feature space showing all the 16 possible item response combinations for the Patient Health Questionnaire–9 items 2 and 4 (phq2&4) instrument. phq2: Patient Health Questionnaire–9 item 2, phq4: Patient Health Questionnaire–9 item 4.

**Figure 2:**
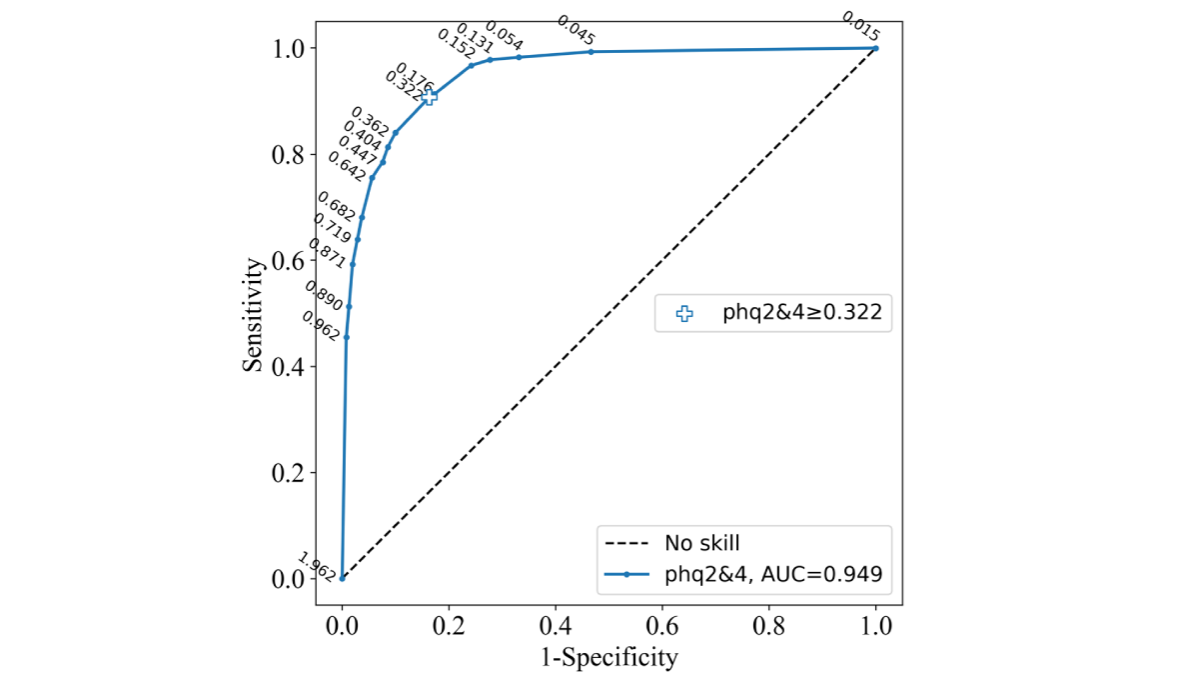
Probability thresholds on the Patient Health Questionnaire–9 items 2 and 4 (phq2&4) machine learning receiver operating characteristic curve on the PROACTIVE training set. Each threshold represents a decision boundary in the input feature space. AUC: area under the curve.

The input feature space for the phq2&4 (Figure 1) contains each combination of *depressed mood* (phq2) and *lack of energy* (PHQ-9 item 4; phq4) responses. The Likert scale 0 to 3 response options for phq2 are on the x-axis and those for phq4 are on the y-axis. Each response combination has an estimated probability of belonging to the screening-positive class. For example, if an individual responded 0 (“Not at all”) to both phq2 and phq4 (bottom left of the input feature space), the assigned probability of that individual being screen positive is 0.015 according to the phq2&4 ML model. These probabilities represent the 16 candidate probability thresholds of the phq2&4 instrument observed on the ROC curve (Figure 2).

Similar to the psychometric measure thresholds, a greater-than-or-equal-to threshold was applied to these probabilities. The performance of the model could be adjusted by varying the applied threshold. As the sensitivity and specificity can be determined from the ROC curve (Figure 2), a threshold that most closely matches the required sensitivity and specificity performance can be chosen. As reported in the *Methods* section, the probability thresholds applied to the item pairings were chosen to maximize the Youden index. The ≥0.322 probability threshold maximized the Youden index for the phq2&4. An individual will be classified as screen positive by the phq2&4 ML model if the assigned probability of their item response combination is ≥0.322; otherwise, they will be classified as screen negative. The red (screen positive) and blue (screen negative) areas represent the predictions of the phq2&4 ML model with the ≥0.322 probability threshold applied (Figure 1). The white line (the decision boundary) that separates the 2 colored areas represents this probability threshold. The input feature space and probability threshold ROC curve for the phq2&8 are provided in Multimedia Appendix 3.

#### CV Performance

**Figure 3:**
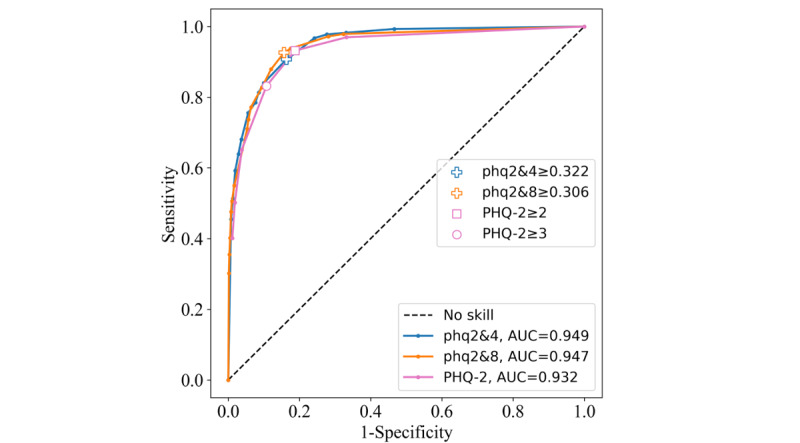
Receiver operating characteristic curves for the Patient Health Questionnaire–9 items 2 and 4 (phq2&4), Patient Health Questionnaire–9 items 2 and 8 (phq2&8), and Patient Health Questionnaire–2 (PHQ-2) instruments on the PROACTIVE training set. AUC: area under the curve.

As found in our previous analysis [12], the phq2&4 and phq2&8 were the best performing, irrespective of removing the oversampling. The phq2&4 and phq2&8 had slightly higher AUCs than the PHQ-2 on CV data (0.949, 0.947, and 0.932, respectively; Figure 3). The reported results are for the maximized CV Youden index thresholds of the phq2&4 and phq2&8 along with the most common PHQ-2 cutoffs of ≥2 and ≥3 (Table 2). The phq2&4 threshold of ≥0.322 achieved a Youden index of 0.744, the highest out of all probability thresholds for the instrument. The PHQ-2≥2 achieved the same Youden index, meaning that their overall performance levels were equivalent despite different sensitivity and specificity statistics.

**Table 2:** Generalization results of Patient Health Questionnaire–2 (PHQ-2), Patient Health Questionnaire–9 items 2 and 4 (phq2&4), and Patient Health Questionnaire–9 items 2 and 8 (phq2&8) instruments on the PROACTIVE data set.

Data set and instrument			Youden index		Sensitivity		Specificity		Positive predictive value		Negative predictive value
**PROACTIVE CV^a^**											
	PHQ-2≥2	0.744		0.932		0.812		0.682		0.965	
	PHQ-2≥3	0.725		0.832		0.893		0.770		0.925	
	phq2&4≥0.322	0.744		0.907		0.837		0.706		0.954	
	phq2&8≥0.306	0.769		0.926		0.843		0.719		0.963	
**PROACTIVE test**											
	PHQ-2≥2	0.753		0.934		0.819		0.690		0.966	
	PHQ-2≥3	0.739		0.849		0.890		0.769		0.932	
	phq2&4≥0.322	0.749		0.915		0.834		0.705		0.958	
	phq2&8≥0.306	0.735		0.907		0.828		0.695		0.954	

^a^CV: cross-validation.

The optimal threshold for the phq2&8 was ≥0.306 with a Youden index of 0.769, which was higher than the phq2&4 threshold and both PHQ-2 cutoffs. The PHQ-2≥3 cutoff had the lowest Youden index (0.725). As expected, the higher ≥3 cutoff on the PHQ-2 is less sensitive and more specific than the ≥2 cutoff. The PHQ-2≥3 cutoff had the highest PPV at 0.770, followed by phq2&8, phq2&4, the PHQ-2≥2 cutoff. The NPVs were higher than the PPVs for all instruments. At 0.965, the best NPV was from the PHQ-2 ≥2 cutoff, slightly better than the phq2&8, which was followed by the phq2&4 and PHQ-2 ≥3 cutoff with the lowest score (Table 2).

#### Test Performance

**Figure 4:**
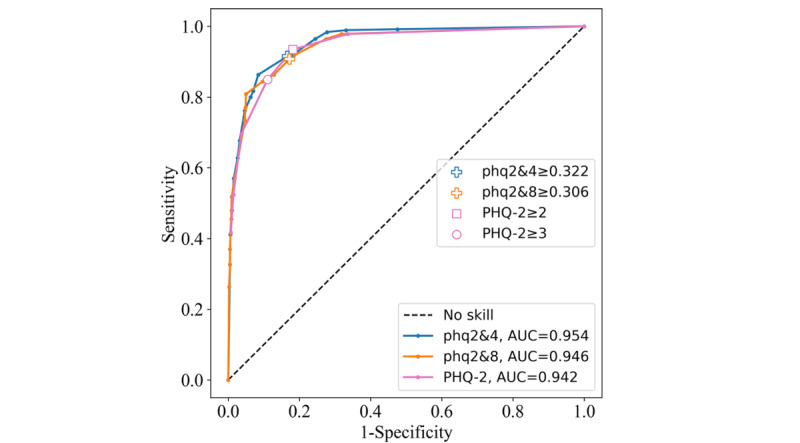
Receiver operating characteristic curves for the Patient Health Questionnaire–9 items 2 and 4 (phq2&4), Patient Health Questionnaire–9 items 2 and 8 (phq2&8), and Patient Health Questionnaire–2 (PHQ-2) instruments on the PROACTIVE test set. AUC: area under the curve.

The new pairings again achieved slightly higher area under the ROC curves than the PHQ-2 on the primary data set’s test split (Figure 4). The phq2&4 yielded 0.954, the phq2&8 yielded 0.946, and the PHQ-2 yielded 0.942. The PHQ-2≥2 cutoff achieved the highest Youden index of 0.753, closely followed by the phq2&4 with 0.749. The PHQ-2≥3 cutoff scored 0.739, whereas the phq2&8 achieved the lowest with 0.735, the largest decrease in CV performance. This lower Youden index for the phq2&8 was a result of a drop in both sensitivity and specificity. Its PPV and NPV were also lower. Metric scores for the phq2&4 and both PHQ-2 cutoffs were similar to those seen in CV (Table 2).

### External Data Sets

#### Performance Overview

The performance of the PHQ-2, phq2&4, and phq2&8 instruments was evaluated on 6 external data sets used to test the generalization of these as prescreening instruments (Table 3). The phq2&4 had the highest AUC on 4 of the 6 data sets: PNS2013, PNS2019, Amazonas, and JC JoyAge. The phq2&8 achieved the highest AUCs on the 2 other data sets, São Paulo-Manaus and MexMedStudents, with the phq2&4 slightly lower but higher than the PHQ-2. The phq2&4 threshold had the highest Youden index on the Amazonas external data set and the joint highest on the PNS2019 external data set. The phq2&8’s threshold scored highest in terms of Youden index on the São Paulo-Manaus and MexMedStudents external data sets. The PHQ-2≥2 cutoff achieved the highest Youden index on the PNS2013 external data set and the joint highest on the PNS2019 external data set. The PHQ-2≥3 cutoff only had the highest score on the JC JoyAge data set, where the ≥2 cutoff was substantially lower. On the MexMedStudents data set, both of the PHQ-2 cutoffs had considerably lower Youden indexes than those of phq2&4 and phq2&8.

**Table 3:** Area under the receiver operating characteristic curve performance across all external data sets used to test the Patient Health Questionnaire–2 (PHQ-2), Patient Health Questionnaire–9 items 2 and 4 (phq2&4), and Patient Health Questionnaire–9 items 2 and 8 (phq2&8) generalization performances.

Instrument	PNS2013^a,^ AUC^b^	PNS2019^c^, AUC	Amazonas, AUC	São Paulo-Manaus, AUC	Mexican Medical Students, AUC	Jockey Club JoyAge, AUC
PHQ-2	0.960	0.961	0.899	0.941	0.838	0.869
phq2&4	0.966	0.969	0.921	0.942	0.879	0.886
phq2&8	0.946	0.949	0.912	0.944	0.884	0.851

^a^PNS2013: Pesquisa Nacional de Saúde 2013.

^b^AUC: area under the curve.

^c^PNS2019: Pesquisa Nacional de Saúde 2019.

#### Brazilian National Health Survey (Pesquisa Nacional de Saúde)

**Figure 5:**
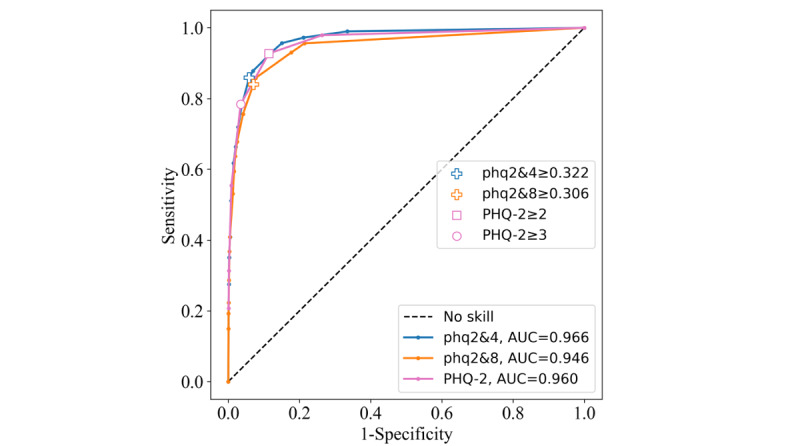
Receiver operating characteristic curves for the Patient Health Questionnaire–9 items 2 and 4 (phq2&4), Patient Health Questionnaire–9 items 2 and 8 (phq2&8), and Patient Health Questionnaire–2 (PHQ-2) instruments on the Pesquisa Nacional de Saúde 2013 data set. AUC: area under the curve.

**Figure 6:**
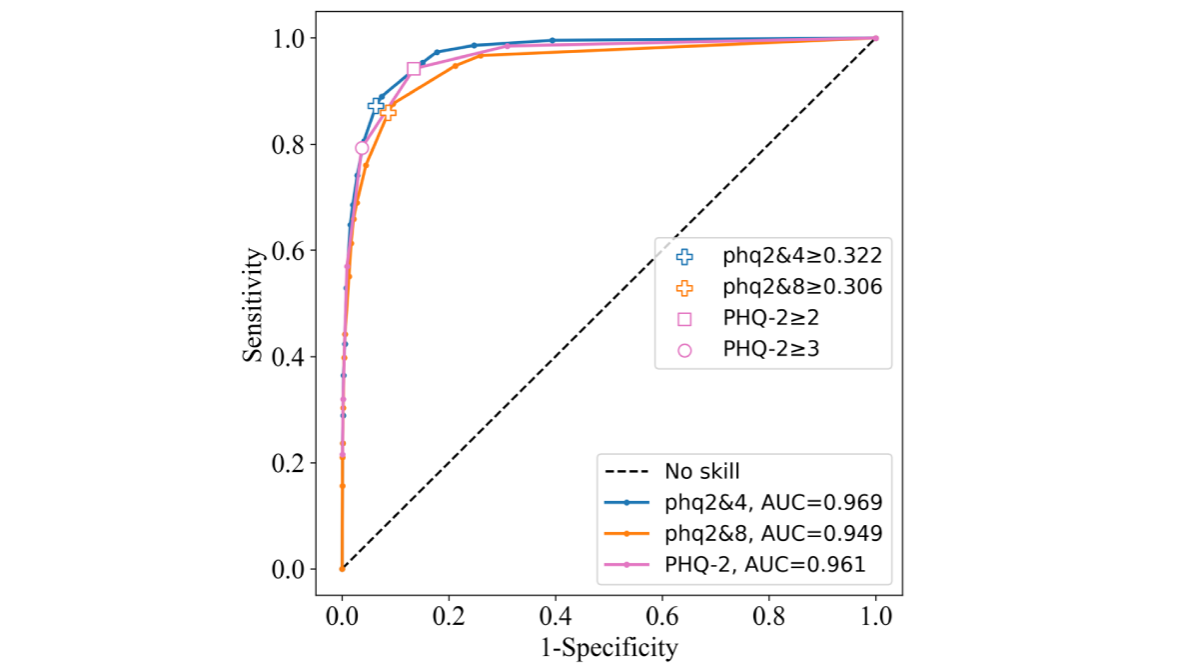
Receiver operating characteristic curves for the Patient Health Questionnaire–9 items 2 and 4 (phq2&4), Patient Health Questionnaire–9 items 2 and 8 (phq2&8), and Patient Health Questionnaire–2 (PHQ-2) instruments on the Pesquisa Nacional de Saúde 2019 data set. AUC: area under the curve.

The ROC curves and AUC performance of each of the instruments were similar across both the PNS2013 (Figure 5) and PNS2019 data sets (Figure 6). The phq2&4 achieved the highest AUC of 0.966 on the PNS2013 data set and 0.969 on the PNS2019 data set. The phq2&8 AUC performance was lower, at 0.946 on the PNS2013 data set and 0.949 on the PNS2019 data set. The PHQ-2 scored lower than phq2&4 but higher than phq2&8, with values of 0.961 and 0.960, respectively. On the PNS2013 data set, the PHQ-2≥2 cutoff outperformed the phq2&4 threshold for Youden index, at 0.813, compared with 0.800. The phq2&8 scored 0.769, and the PHQ-2≥3 cutoff had the lowest score of 0.749. The sensitivity and specificity statistics were high across each instrument’s threshold, with none being overly sensitive or specific. For Youden indices on the PNS2019 data set, the phq2&4 and the PHQ-2≥2 cutoff achieved the same score of 0.808, indicating that they are equally optimal points on the ROC curve despite having different values of sensitivity and specificity (giving equal weight to false positives and false negatives). The phq2&8 achieved a Youden index of 0.772, and the PHQ-2≥3 cutoff scored the lowest with 0.756 (Table 4).

**Table 4:** Generalization results of Patient Health Questionnaire–2 (PHQ-2), Patient Health Questionnaire–9 items 2 and 4 (phq2&4), and Patient Health Questionnaire–9 items 2 and 8 (phq2&8) instruments on the 6 external data sets.

Data set and instrument			Youden index		Sensitivity		Specificity		Positive predictive value		Negative predictive value
**PNS2013^a^**											
	PHQ-2≥2	0.813		0.927		0.886		0.428		0.993	
	PHQ-2≥3	0.749		0.784		0.965		0.674		0.980	
	phq2&4≥0.322	0.800		0.859		0.941		0.572		0.986	
	phq2&8≥0.306	0.769		0.840		0.930		0.522		0.984	
**PNS2019^b^**											
	PHQ-2≥2	0.808		0.942		0.866		0.450		0.992	
	PHQ-2≥3	0.756		0.793		0.963		0.716		0.976	
	phq2&4≥0.322	0.808		0.872		0.937		0.616		0.984	
	phq2&8≥0.306	0.772		0.859		0.914		0.537		0.982	
**Amazonas**											
	PHQ-2≥2	0.656		0.891		0.765		0.474		0.967	
	PHQ-2≥3	0.640		0.751		0.889		0.617		0.938	
	phq2&4≥0.322	0.684		0.834		0.850		0.569		0.956	
	phq2&8≥0.306	0.674		0.827		0.847		0.562		0.954	
**São Paulo-Manaus**											
	PHQ-2≥2	0.766		0.906		0.860		0.375		0.990	
	PHQ-2≥3	0.755		0.821		0.934		0.536		0.982	
	phq2&4≥0.322	0.719		0.829		0.890		0.411		0.982	
	phq2&8≥0.306	0.776		0.889		0.887		0.423		0.989	
**Mexican Medical Students**											
	PHQ-2≥2	0.463		0.906		0.557		0.403		0.947	
	PHQ-2≥3	0.492		0.557		0.935		0.738		0.865	
	phq2&4≥0.322	0.623		0.755		0.868		0.653		0.915	
	phq2&8≥0.306	0.649		0.781		0.868		0.661		0.923	
**Jockey Club** **JoyAge**											
	PHQ-2≥2	0.452		0.958		0.494		0.310		0.980	
	PHQ-2≥3	0.595		0.822		0.773		0.462		0.948	
	phq2&4≥0.322	0.590		0.915		0.675		0.400		0.971	
	phq2&8≥0.306	0.535		0.841		0.695		0.395		0.948	

^a^PNS2013: Pesquisa Nacional de Saúde 2013.

^b^PNS2019 Pesquisa Nacional de Saúde 2019.

#### Amazonas

**Figure 7:**
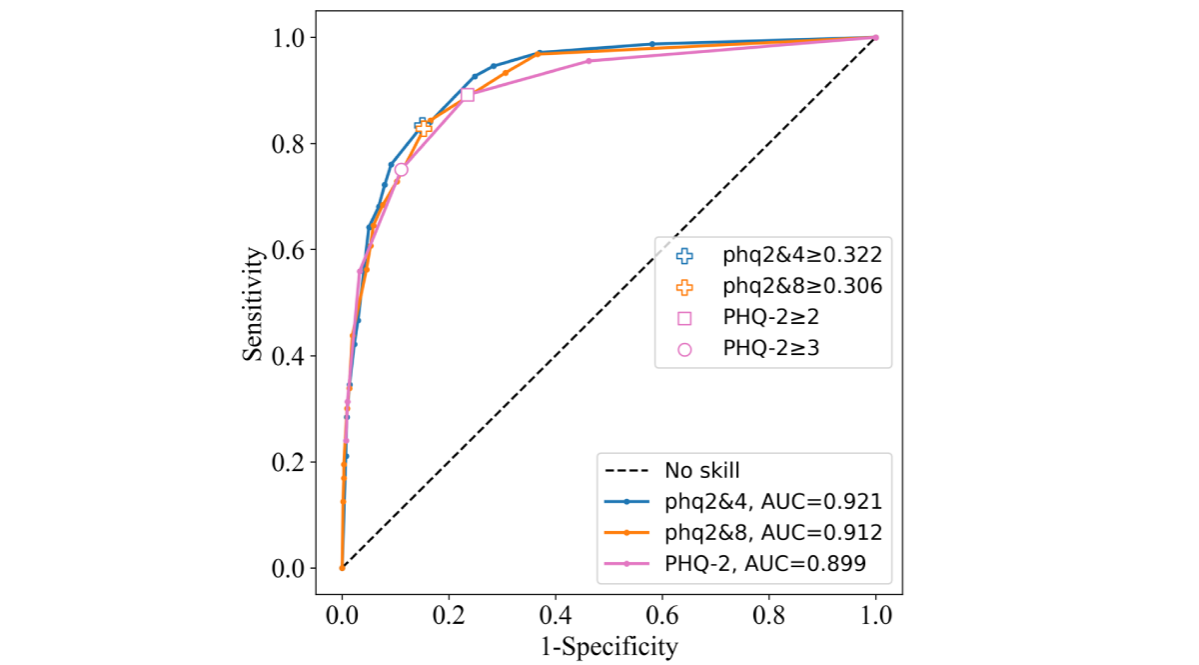
Receiver operating characteristic curves for the Patient Health Questionnaire–9 items 2 and 4 (phq2&4), Patient Health Questionnaire–9 items 2 and 8 (phq2&8), and Patient Health Questionnaire–2 (PHQ-2) instruments on the Amazonas data set. AUC: area under the curve.

The phq2&4 and phq2&8 had the highest AUCs: 0.921 and 0.912, respectively. The phq2&4 threshold scored the highest for Youden index (0.684), and the phq2&8 threshold scored 0.674. The PHQ-2 achieved the lowest AUC of 0.899, and both the ≥2 and ≥3 cutoffs’ Youden indexes were lower, at 0.656 and 0.640, respectively. Again, the sensitivity and specificity were high across the board for all the instruments. The ROC curves on the Amazonas data set (Figure 7) show superior ROC performance of the phq2&4 and phq2&8 compared with the PHQ-2, having higher AUC values and thresholds located closer to the optimal (top left) point of the graph.

#### São Paulo-Manaus

**Figure 8:**
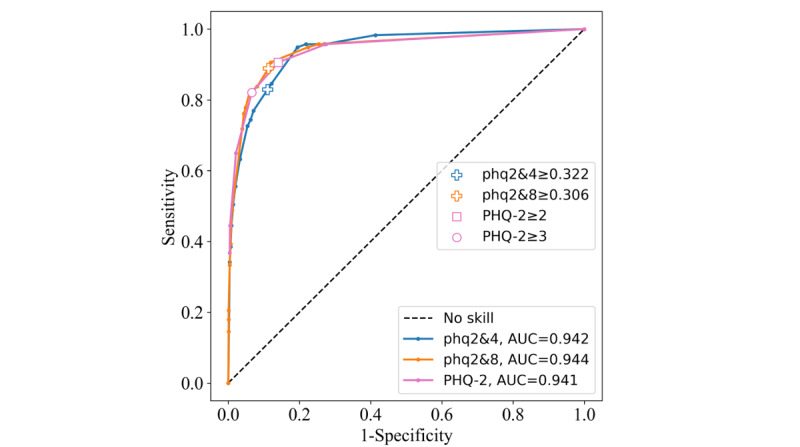
Receiver operating characteristic curves for the Patient Health Questionnaire–9 items 2 and 4 (phq2&4), Patient Health Questionnaire–9 items 2 and 8 (phq2&8), and Patient Health Questionnaire–2 (PHQ-2) instruments on the São Paulo-Manaus data set. AUC: area under the curve.

The phq2&8 was the best instrument in this sample, with an AUC of 0.944 and a Youden index of 0.776 for its threshold. The phq2&4 achieved a slightly lower AUC of 0.942, but its threshold was the poorest in this sample, with a Youden index of 0.719. This is evident when observing the phq2&4 threshold of ≥0.322, which falls below the ROC curves of the other instruments (Figure 8). The PHQ-2 AUC was marginally lower (0.941). The ≥2 cutoff outperformed the ≥3 cutoff with a Youden index of 0.766 compared with 0.755. Again, no threshold or cutoff was overly sensitive or specific in this sample (Table 4).

#### Mexican Medical Students

**Figure 9:**
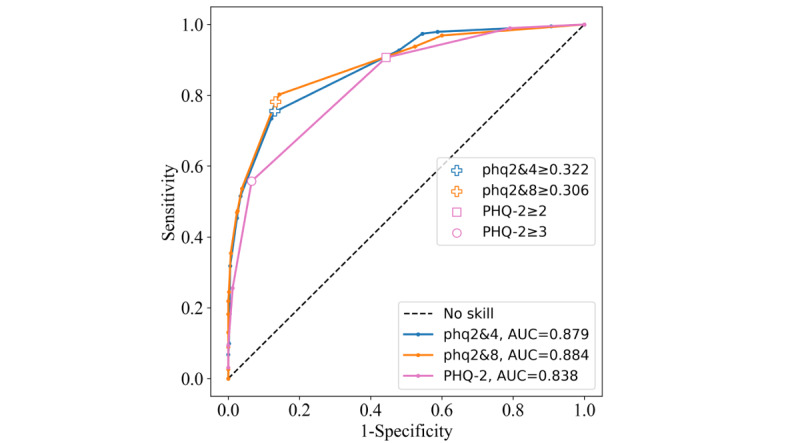
Receiver operating characteristic curves for the Patient Health Questionnaire–9 items 2 and 4 (phq2&4), Patient Health Questionnaire–9 items 2 and 8 (phq2&8), and Patient Health Questionnaire–2 (PHQ-2) instruments on the Mexican Medical Students data set. AUC: area under the curve.

The performance gap between the new phq2&4 and phq2&8 instruments and the PHQ-2 was the largest in this data set. The ROC curves displayed this gap in terms of shape, AUC, and threshold and cutoff locations (Figure 9). The phq2&4 and phq2&8 instruments achieved higher AUC values of 0.879 and 0.884, respectively, compared with 0.838 for the PHQ-2. The phq2&4 ≥0.322 and phq2&8 ≥0.322 thresholds generalized well, with Youden indices of 0.623 and 0.649, respectively. Both PHQ-2 cutoffs performed poorly in this sample: the ≥2 cutoff was highly sensitive with low specificity, and ≥3 was highly specific with low sensitivity. This resulted in poor combined performance, as seen with Youden indices of 0.463 and 0.492 for the PHQ-2≥2 and PHQ-2≥3 cutoffs, respectively (Table 4). This was the first data set in which the ≥2 cutoff performed worse than the ≥3 cutoff.

#### JC JoyAge

**Figure 10:**
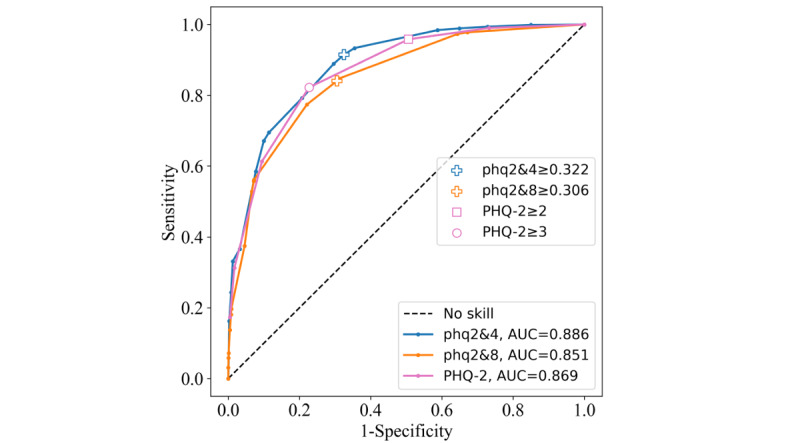
Receiver operating characteristic curves for the Patient Health Questionnaire–9 items 2 and 4 (phq2&4), Patient Health Questionnaire–9 items 2 and 8 (phq2&8), and Patient Health Questionnaire–2 (PHQ-2) instruments on the Jockey Club JoyAge data set. AUC: area under the curve.

The phq2&4 achieved the highest AUC in this sample (0.886). The performance of the phq2&8 dropped with an AUC of 0.851, whereas the PHQ-2 achieved 0.869 (Figure 10). The PHQ-2≥3 cutoff had the highest Youden index (0.595), again outperforming the ≥2 cutoff. The PHQ-2≥2 cutoff performance was highly sensitive but poorly specific, resulting in a low Youden index of 0.452. The phq2&4 and phq2&8 thresholds scored 0.590 and 0.535, respectively, for Youden index (Table 4).

## Discussion

### Principal Findings

To avoid selective outcome reporting in threshold results, the optimal thresholds of the phq2&4 and phq2&8 instruments were based on the highest CV Youden index during the model training process. Only the optimal thresholds of the phq2&4 and phq2&8 instruments, ≥0.322 and ≥0.306, respectively, were reported, but both the common PHQ-2 cutoffs, ≥2 and ≥3, were reported. All cutoffs and threshold performance scores for the PHQ-2 psychometric measure method and phq2&4 and phq2&8 ML method are provided in Multimedia Appendix 2.

The phq2&4 instrument generalized best, having the highest AUC on the PROACTIVE test set and in 4 of the 6 external data sets (range 0.879-0.969). It achieved a higher AUC than the PHQ-2 on all data sets and was only outperformed by the phq2&8 on 2 of the external data sets. The phq2&4 threshold had the highest Youden index on the Amazonas data set and was joint highest on the PNS2019 data set with the PHQ-2. However, it was the most reliable across all sets, with the narrowest Youden index range (range 0.590-0.908). The phq2&4’s performance waned most compared with the other instruments on the São Paulo-Manaus data set with a Youden index well below that of the phq2&8 and the 2 PHQ-2 cutoffs. Despite this, the phq2&4 AUC was marginally higher than that of the PHQ-2. The phq2&8 scored highest in terms of AUC and Youden index on the São Paulo-Manaus and MexMedStudents external data sets. Its AUC was lower than that of the PHQ-2 on the PNS2013, PNS2019, and JC JoyAge external data sets. However, overall, the phq2&8 fluctuated less on both AUC (range 0.851-0.949) and Youden index (range 0.535-0.776).

The PHQ-2 did not achieve the highest AUC in any of the data sets evaluated. Its performance also fluctuated more than the phq2&4 and phq2&8 on the external data sets (range 0.838-0.961). The PHQ-2’s worst performance was on the MexMedStudents data set with a substantially lower AUC than the phq2&4 and phq2&8. Here, both the ≥2 and ≥3 cutoffs of the PHQ-2 also had considerably lower Youden indices than the phq2&4 and phq2&8. Both PHQ-2 cutoffs showed variable performance across the various external data sets. Their performance levels fluctuated more than the phq2&4 and phq2&8 thresholds seen with the broader range of Youden indexes from the ≥2 (range 0.452-0.813) and ≥3 (range 0.492-0.755) cutoffs.

The PHQ-2≥2 cutoff achieved the highest Youden index on 1 of the external data sets presented, PNS2013, and achieved the joint highest on PNS2019. Interestingly, the PHQ-2≥3 achieved the highest Youden index on the JC JoyAge data set, whereas the ≥2 cutoff scored substantially lower in this sample. The ≥3 cutoff also had a higher Youden index on the MexMedStudents data set. Arrieta et al [25] also found that ≥3 was the optimal cutoff in a Mexican cohort when using PHQ-9 scores ≥10 as the reference standard. This highlights the uncertainty around the optimal cutoff, which may be specific to certain populations and may be unknown before administering it as a prescreening instrument. The higher NPV than PPV seen from all instruments supports the view that ultrabrief questionnaires are better suited as “rule-out” instruments, where a prescreen negative strongly suggests the absence of depressive symptoms [9]. Nonetheless, the findings may differ depending on the probability threshold or the cutoff applied.

The weakest performance for all 3 instruments was observed on the MexMedStudents and JC JoyAge data sets, which were the only non-Brazilian data sets used in this analysis. The JC JoyAge data set represented a different cultural context in Hong Kong but had a similar age demographic to the Brazilian primary data set, PROACTIVE. Conversely, the MexMedStudents data set consisted of younger and more educated individuals from Mexico. The comparatively lower performance of the phq2&4 and phq2&8 on these 2 non-Brazilian data sets, in contrast to the other Brazilian data sets, may be attributed to the fact that these pairings were identified within the Brazilian PROACTIVE data set. Despite the phq2&4’s AUC being lower on these 2 data sets compared with the other 4 external data sets, it still outperformed the PHQ-2 on both data sets. Meanwhile, the phq2&8 performed better than the PHQ-2 on the MexMedStudents data set but worse on the JC JoyAge data set. The PHQ-2 was developed in a Western population, which may explain its lower AUC performance on these data sets collected in different populations. Nevertheless, these results highlight the PHQ-2’s limited effectiveness in prescreening for depressive symptoms in the various cultures and demographics analyzed in this study.

The superior AUC performance of the phq2&4, albeit only slightly in some data sets, suggests that the *low-energy* item merits inclusion in ultrabrief questionnaires for the prescreening of depressive symptomatology. In terms of AUC, the phq2&8 did not generalize as well. However, its threshold achieved the highest Youden index on multiple data sets. This suggests that the *psychomotor disturbances* item also merits consideration for inclusion in prescreening instruments. Symptoms of fatigue and psychomotor dysfunction may have been overlooked despite evidence suggesting their increased importance in the diagnosis of MDD [26].

### Limitations

A limitation of this study was the use of PHQ-9 scores of ≥10 as the reference standard and not a clinical diagnosis from an interview such as the Composite International Diagnostic Interview, Mini-International Neuropsychiatric Interview, or Structured Clinical Interview for DSM-V. Ultrabrief questionnaires for depressive symptoms are intended for use as part of a 2-stage screening process and should be followed by a more in-depth measure, such as the PHQ-9, following a prescreen positive result [4]. Therefore, it could be argued that the primary role of a depressive symptomatology prescreening instrument is to optimally identify individuals who will score ≥10 on the full PHQ-9, a common entry requirement for depression clinical trials, and not to directly predict depression. Nevertheless, before either the phq2&4 or phq2&8 are administered as prescreening instruments, their performance must be evaluated with an MDD clinical diagnosis used as the reference standard. Alternative optimal ultrabrief questionnaires may emerge when performance is evaluated against a clinical diagnosis.

Potential result biases could arise from the fact that 4 out of the 6 external data sets used in this study were from Brazil, the same country as the primary data set from which the optimal pairings were selected. However, it is important to note that these external data sets encompassed diverse populations within Brazil, including different states and age groups. The remaining 2 external data sets were collected in Mexico and Hong Kong, with the former consisting of younger medical school students and the latter consisting of individuals with an age profile similar to the PROACTIVE data set. The selection of the phq2&4 and phq2&8 was solely based on their performance on the primary data set. Their performance on 6 external data sets, without any model retraining, did not influence their selection as the best pairings. This minimized the instrument selection biases and enhanced the validity of the findings.

In a similar vein, another limitation of the study was that the data sets used were all from non-Western populations. This could also be considered a strength, as Western populations have been overrepresented in previous studies and meta-analyses [27]. Therefore, our findings increase the availability of studies from alternative populations. Further exploration of pairing performance in data sets from different cultures and where the PHQ-9 was administered in languages and dialects other than those evaluated here is required to validate the reliability of the newly found optimal pairings. It also remains essential to assess the generalizability of the newly found pairing in Western populations to evaluate pairing performance in the demographic in which the PHQ-2 was established. To examine reproducibility rather than generalizability, new ML models would need to be trained on each data set evaluated. This may lead to different pairing results, with unique optimal pairings found for each sample. This would suggest each population might require their own depressive symptomatology classification instrument, and a global optimal ultrabrief questionnaire may not exist. However, this approach can lead to overfitting when the instruments are too specialized and only suitable for the particular sample in which it was found.

The strong generalization performance of the phq2&4 and phq2&8 compared with the PHQ-2 on the 6 external data sets, despite being trained on 1 data set, indicated that they are more suitable as global ultrabrief questionnaires. Before administering either the phq2&4 or phq2&8 as prescreening instruments, further investigation is required into the impact item order has on the PHQ-9 and depressive symptomatology screening outcomes. The effect item order has on how a respondent endorses an item, on the neighboring items, and on the PHQ-9 sum scores is unknown. The use of either of these newly proposed prescreening instruments could affect item responses, and the outcome of the full PHQ-9, given items would be skipped. Future research involving a split test (A/B test) with different PHQ-9 item orders or with the PHQ-2 versus the phq2&4 for prescreening would help narrow this knowledge gap.

### Conclusions

A re-examination of the cardinal symptoms of depression has been suggested to maintain the high standards required for good clinical practice [26]. Depression symptoms are rarely evaluated when combined as pairings, and when they are, performance levels are so similar that the selection of the best symptom pairing is considered somewhat arbitrary [11]. The objective of this study was to use a data-driven ML approach to identify and validate the most predictive 2-item depressive symptomatology ultrabrief questionnaire from the 9 items that comprise the PHQ-9. ML algorithms have been previously used to develop brief versions of parent questionnaires by identifying the most significant predictors [28].

In this analysis, the PHQ-2 has not demonstrated its superiority as a prescreening instrument when compared with other item pairings within the PHQ-9 item set. Comparing the performance of all 36 pairings gave an equal opportunity to each pairing and avoided any selection bias of the best 2-item ultrabrief questionnaire. Solely looking at individual performance when constructing ultrabrief questionnaires for depressive symptoms may lead to suboptimal performance as context on how items interrelate could be overlooked. Compared with the alternatives, the *anhedonia* item underperformed when paired with the *depressed mood* item. This suggests that it may be an arbitrary choice as a partner for the *depressed mood* item in prescreening instruments. *Anhedonia*, which involves a lack of pleasure rather than overt sadness, has been associated with higher levels of depression severity [29]. The inclusion of an *anhedonia* item in ultrabrief screening questionnaires, instead of potentially superior alternatives, may result in failure to identify individuals with moderate levels of depression severity.

The idea that cognitive symptoms play a more significant role in the diagnosis of depression is primarily a concept of the 20th-century Western world and may not hold true in other cultures to the same extent [29]. Instead, pairing the *depressed mood* item with the *low-energy* item (phq2&4) or the *psychomotor agitation or retardation* item (phq2&8) achieved higher AUC statistics on the primary data set. The phq2&4 and phq2&8 pairings include 1 cognitive symptom in *depressed mood*, 1 of the so-called cardinal symptoms of depression, alongside a neurovegetative symptom *in low energy* or *psychomotor changes*. This combination may be more beneficial in detecting the broad range of symptom profiles instead of asking 2 cognitive symptoms.

To further delve into these new pairings, their performance was investigated on 6 external data sets from 6 separate studies and with different participant demographics. These external data sets were used purely as test sets, meaning the ML models were not retrained and so did not learn any new information on how to best classify screen positive and screen negative samples in these data sets. Therefore, this analysis tested the generalizability of the phq2&4 and phq2&8 rather than their reproducibility across multiple data sets. Prevalence was not controlled for in this analysis, which enabled a more complete evaluation of the phq2&4 and phq2&8 generalization performance. This determined whether these new pairings and their respective ML models trained on an older adult, socioeconomically deprived Brazilian population generalized well and could be used as depressive symptomatology prescreening instruments for clinical trial recruitment in different demographics in place of the PHQ-2.

Combining item pairings with ML models allowed for a more flexible approach to the classification of depressive symptomatology. The summation and greater-than-or-equal-to logic of psychometric measures such as the PHQ-2 place equal weighting on each item. This approach has been questioned both theoretically and empirically [30]. ML models are not limited to this summation and greater-than-or-equal-to logic. This is possible because the LR coefficients allow for different weighting of items depending on their learned importance in the classification of depressive symptomatology. Hence, ML models have more thresholds to choose from (16 compared with 7 of the PHQ-2) to fine-tune performance until a desirable classification threshold is achieved.

Previously, the use of LR in conjunction with a screening questionnaire was considered too complex, as the presence of depressive symptomatology could not be identified quickly or by hand owing to the different weighting of the items [31]. However, given the drastic improvement in technology and the increased use of technical devices to collect data in clinical trials [17], the use of LR for screening is more viable nowadays. ML models can be deployed within screening applications where interviewers input patient responses and receive predictions on the presence of depressive symptomatology (prescreen positive or negative).

During this analysis, it was assumed that the optimal thresholds of the phq2&4 and phq2&8 should be determined using the maximum Youden indices. This may not be a suitable threshold for all prescreening contexts, especially in applications where instruments may be required to be more sensitive or more specific [32]. However, as the most suitable threshold is situational, both sensitivity and specificity were weighted equally using the Youden index. The optimal pairings were ranked on AUC, which measures the performance across all thresholds of an instrument, rather than the performance of the optimal threshold. A higher AUC indicates a minimal performance impact when adjusting the instrument’s threshold. Having a greater number of thresholds to tune classification performance is another advantage that combining the PHQ-9 item pairings with ML models has over the summation and greater-than-or-equal-to logic of the psychometric approach.

An instrument’s threshold or cutoff must be selected before its use. With the PHQ-2’s most common cutoffs showing fluctuating performance, its use as a prescreening instrument could result in a larger number of misclassifications if a suboptimal threshold was chosen. The consistency of the findings for the phq2&4 and phq2&8 across multiple external data sets suggests that the strong performance seen is not by chance. These new symptom pairings warrant further investigation into how well they perform as prescreening instruments for depressive symptomatology in various populations.

## Appendix

Multimedia Appendix 1 Hyperparameters and multiple linear regression equations of the Patient Health Questionnaire–9 items 2 and 4 and Patient Health Questionnaire–9 items 2 and 8 logistic regression models.

Multimedia Appendix 2 Tables containing all threshold scores for the Patient Health Questionnaire–9 items 2 and 4, Patient Health Questionnaire–9 items 2 and 8, and Patient Health Questionnaire–2 on each data set.

Multimedia Appendix 3 The input feature space and probability threshold receiver operating characteristic curve for the Patient Health Questionnaire–9 items 2 and 8.

## Abbreviations

AUC: area under the curve
CV: cross-validation
DSM-V: Diagnostic and Statistical Manual of Mental Disorders, fifth edition
JC: Jockey Club
LR: logistic regression
MDD: major depressive disorder
MexMedStudents: Mexican Medical Students
ML: machine learning
NPV: negative predictive value
PHQ: Patient Health Questionnaire
phq2&4: Patient Health Questionnaire–9 items 2 and 4
phq2&8: Patient Health Questionnaire–9 items 2 and 8
phq2: Patient Health Questionnaire–9 item 2
phq4: Patient Health Questionnaire–9 item 4
PNS2013: Pesquisa Nacional de Saúde 2013
PNS2019: Pesquisa Nacional de Saúde 2019
PPV: positive predictive value
ROC: receiver operating characteristic
