# Supplementary material for: Patient Health Questionnaire-9 Item Pairing Predictiveness for Prescreening Depressive Symptomatology: Machine Learning Analysis
Source: JMIR Ment Health. 2023 Oct 19;10:e48444. doi: 10.2196/48444 (PMC10623235; doi:10.2196/48444)

## Multimedia Appendix 1. Hyperparameters and multiple linear regression equations of the phq2&4 and phq2&8 logistic regression models

### Phq2&4

Hyper-parameters:

C = 0.00316

Penalty = L2

Tolerance = 0.0001

Solver = Limited-memory Broyden-Fletcher-Goldfarb-Shanno algorithm (LBFGS)

Multiple linear regression equation:


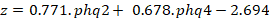


### Phq2&8

Hyper-parameters:

C = 0.00320

Penalty = L2

Tolerance = 0.0001

Solver = Limited-memory Broyden-Fletcher-Goldfarb-Shanno algorithm (LBFGS)

Multiple linear regression equation:


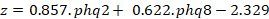

Supplement: Multimedia Appendix 1 [file mental_v10i1e48444_app1.doc]
