# Supplementary material for: Patient Health Questionnaire-9 Item Pairing Predictiveness for Prescreening Depressive Symptomatology: Machine Learning Analysis
Source: JMIR Ment Health. 2023 Oct 19;10:e48444. doi: 10.2196/48444 (PMC10623235; doi:10.2196/48444)
Supplement: Multimedia Appendix 2 [file mental_v10i1e48444_app2.doc]

Table S1 – Cross-validation Area Under the Curve (AUC) scores for each ML-based pairing instrument, from phq1&2 to phq8&9, on the primary dataset (PROACTIVE).

| Pairing | AUC | Optimal  Threshold | Youden | Sens | Spec | PPV | NPV |
| --- | --- | --- | --- | --- | --- | --- | --- |
|  |  |  |  |  |  |  |  |
| phq1&2 | 0.936 | 0.244 | 0.744 | 0.932 | 0.812 | 0.683 | 0.965 |
| phq1&3 | 0.922 | 0.393 | 0.676 | 0.768 | 0.908 | 0.783 | 0.901 |
| phq1&4 | 0.931 | 0.350 | 0.717 | 0.884 | 0.834 | 0.697 | 0.943 |
| phq1&5 | 0.909 | 0.251 | 0.687 | 0.868 | 0.819 | 0.676 | 0.935 |
| phq1&6 | 0.926 | 0.344 | 0.739 | 0.878 | 0.861 | 0.732 | 0.942 |
| phq1&7 | 0.913 | 0.307 | 0.717 | 0.861 | 0.856 | 0.721 | 0.934 |
| phq1&8 | 0.925 | 0.315 | 0.716 | 0.866 | 0.850 | 0.715 | 0.936 |
| phq1&9 | 0.883 | 0.230 | 0.657 | 0.884 | 0.774 | 0.629 | 0.939 |
| phq2&3 | 0.942 | 0.281 | 0.750 | 0.859 | 0.891 | 0.775 | 0.936 |
| phq2&4 | 0.949 | 0.322 | 0.744 | 0.907 | 0.837 | 0.706 | 0.954 |
| phq2&5 | 0.938 | 0.273 | 0.745 | 0.911 | 0.835 | 0.705 | 0.956 |
| phq2&6 | 0.929 | 0.250 | 0.744 | 0.887 | 0.857 | 0.729 | 0.946 |
| phq2&7 | 0.939 | 0.325 | 0.766 | 0.891 | 0.875 | 0.755 | 0.949 |
| phq2&8 | 0.947 | 0.306 | 0.769 | 0.926 | 0.843 | 0.719 | 0.963 |
| phq2&9 | 0.909 | 0.295 | 0.702 | 0.792 | 0.910 | 0.793 | 0.910 |
| phq3&4 | 0.927 | 0.261 | 0.719 | 0.894 | 0.825 | 0.690 | 0.947 |
| phq3&5 | 0.875 | 0.273 | 0.635 | 0.854 | 0.780 | 0.627 | 0.925 |
| phq3&6 | 0.910 | 0.296 | 0.671 | 0.871 | 0.800 | 0.654 | 0.935 |
| phq3&7 | 0.885 | 0.269 | 0.652 | 0.887 | 0.765 | 0.621 | 0.940 |
| phq3&8 | 0.902 | 0.279 | 0.662 | 0.915 | 0.747 | 0.611 | 0.953 |
| phq3&9 | 0.845 | 0.233 | 0.606 | 0.803 | 0.804 | 0.639 | 0.904 |
| phq4&5 | 0.920 | 0.360 | 0.709 | 0.846 | 0.863 | 0.728 | 0.928 |
| phq4&6 | 0.932 | 0.471 | 0.731 | 0.859 | 0.872 | 0.744 | 0.935 |
| phq4&7 | 0.929 | 0.414 | 0.711 | 0.853 | 0.858 | 0.723 | 0.931 |
| phq4&8 | 0.931 | 0.199 | 0.708 | 0.944 | 0.765 | 0.634 | 0.969 |
| phq4&9 | 0.904 | 0.364 | 0.696 | 0.866 | 0.830 | 0.688 | 0.935 |
| phq5&6 | 0.890 | 0.324 | 0.663 | 0.803 | 0.860 | 0.713 | 0.910 |
| phq5&7 | 0.858 | 0.372 | 0.625 | 0.771 | 0.854 | 0.696 | 0.896 |
| phq5&8 | 0.883 | 0.315 | 0.652 | 0.820 | 0.832 | 0.680 | 0.915 |
| phq5&9 | 0.807 | 0.282 | 0.549 | 0.737 | 0.812 | 0.630 | 0.877 |
| phq6&7 | 0.884 | 0.418 | 0.677 | 0.776 | 0.901 | 0.773 | 0.903 |
| phq6&8 | 0.897 | 0.346 | 0.691 | 0.813 | 0.878 | 0.743 | 0.916 |
| phq6&9 | 0.829 | 0.250 | 0.605 | 0.734 | 0.870 | 0.713 | 0.883 |
| phq7&8 | 0.872 | 0.332 | 0.643 | 0.772 | 0.871 | 0.721 | 0.898 |
| phq7&9 | 0.797 | 0.331 | 0.540 | 0.680 | 0.859 | 0.677 | 0.861 |
| phq8&9 | 0.834 | 0.275 | 0.600 | 0.773 | 0.827 | 0.659 | 0.894 |

Table S2.1 – Cross-Validation results of PHQ-2 instrument for all discrete cutpointss on the primary dataset (PROACTIVE).

|  |  | PROACTIVE CV | | | | |
| --- | --- | --- | --- | --- | --- | --- |
| Pairing | Cutpoint | Youden | Sens | Spec | PPV | NPV |
| PHQ-2 | ≥0 | 0 | 1 | 0 | 0.302 | 0 |
|  | ≥1 | 0.638 | 0.969 | 0.668 | 0.559 | 0.981 |
|  | ≥2 | 0.744 | 0.932 | 0.812 | 0.682 | 0.965 |
|  | ≥3 | 0.725 | 0.832 | 0.893 | 0.77 | 0.925 |
|  | ≥4 | 0.613 | 0.651 | 0.962 | 0.882 | 0.864 |
|  | ≥5 | 0.483 | 0.502 | 0.982 | 0.922 | 0.82 |
|  | ≥6 | 0.39 | 0.401 | 0.989 | 0.939 | 0.792 |

Table S2.2 – Cross-Validation results of phq2&4 instrument for all probability thresholds on the primary dataset (PROACTIVE).

|  |  | PROACTIVE CV | | | | |
| --- | --- | --- | --- | --- | --- | --- |
| Pairing | Threshold | Youden | Sens | Spec | PPV | NPV |
| phq2&4 | ≥0.015 | 0 | 1 | 0 | 0.302 | 0 |
|  | ≥0.045 | 0.527 | 0.993 | 0.534 | 0.48 | 0.994 |
|  | ≥0.054 | 0.652 | 0.982 | 0.669 | 0.563 | 0.989 |
|  | ≥0.131 | 0.701 | 0.978 | 0.723 | 0.605 | 0.987 |
|  | ≥0.152 | 0.725 | 0.967 | 0.758 | 0.634 | 0.982 |
|  | ≥0.176 | 0.74 | 0.913 | 0.827 | 0.696 | 0.956 |
|  | ≥0.322 | 0.744 | 0.907 | 0.837 | 0.706 | 0.954 |
|  | ≥0.362 | 0.74 | 0.84 | 0.9 | 0.785 | 0.929 |
|  | ≥0.404 | 0.728 | 0.813 | 0.915 | 0.805 | 0.919 |
|  | ≥0.447 | 0.709 | 0.785 | 0.924 | 0.818 | 0.908 |
|  | ≥0.642 | 0.7 | 0.756 | 0.944 | 0.854 | 0.899 |
|  | ≥0.682 | 0.644 | 0.68 | 0.963 | 0.889 | 0.874 |
|  | ≥0.719 | 0.611 | 0.639 | 0.972 | 0.907 | 0.862 |
|  | ≥0.871 | 0.573 | 0.592 | 0.981 | 0.93 | 0.847 |
|  | ≥0.89 | 0.5 | 0.512 | 0.987 | 0.946 | 0.824 |
|  | ≥0.962 | 0.447 | 0.455 | 0.992 | 0.963 | 0.808 |

Table S2.3 – Cross-Validation results of phq2&8 instrument for all probability thresholds on the primary dataset (PROACTIVE).

|  |  | PROACTIVE CV | | | | |
| --- | --- | --- | --- | --- | --- | --- |
| Pairing | Threshold | Youden | Sens | Spec | PPV | NPV |
| phq2&8 | ≥0.129 | 0 | 1 | 0 | 0.302 | 0 |
|  | ≥0.19 | 0.655 | 0.979 | 0.677 | 0.567 | 0.987 |
|  | ≥0.217 | 0.691 | 0.972 | 0.719 | 0.6 | 0.983 |
|  | ≥0.272 | 0.763 | 0.938 | 0.825 | 0.699 | 0.968 |
|  | ≥0.306 | 0.769 | 0.926 | 0.843 | 0.719 | 0.963 |
|  | ≥0.342 | 0.759 | 0.879 | 0.88 | 0.76 | 0.944 |
|  | ≥0.371 | 0.733 | 0.827 | 0.906 | 0.792 | 0.924 |
|  | ≥0.411 | 0.708 | 0.772 | 0.936 | 0.84 | 0.905 |
|  | ≥0.452 | 0.68 | 0.737 | 0.944 | 0.85 | 0.892 |
|  | ≥0.493 | 0.657 | 0.71 | 0.947 | 0.853 | 0.883 |
|  | ≥0.525 | 0.533 | 0.55 | 0.983 | 0.934 | 0.835 |
|  | ≥0.566 | 0.495 | 0.505 | 0.99 | 0.956 | 0.822 |
|  | ≥0.607 | 0.468 | 0.476 | 0.992 | 0.964 | 0.814 |
|  | ≥0.674 | 0.397 | 0.402 | 0.995 | 0.972 | 0.794 |
|  | ≥0.71 | 0.352 | 0.355 | 0.997 | 0.981 | 0.781 |
|  | ≥0.795 | 0.3 | 0.302 | 0.998 | 0.988 | 0.768 |

Table S3.1 – Test results of PHQ-2 instrument for all discrete cutpoints on the primary dataset (PROACTIVE).

|  |  | PROACTIVE Test | | | | |
| --- | --- | --- | --- | --- | --- | --- |
| Pairing | Cutpoint | Youden | Sens | Spec | PPV | NPV |
| PHQ-2 | ≥0 | 0 | 1 | 0 | 0.302 | 0 |
|  | ≥1 | 0.642 | 0.978 | 0.664 | 0.558 | 0.986 |
|  | ≥2 | 0.753 | 0.934 | 0.819 | 0.69 | 0.966 |
|  | ≥3 | 0.739 | 0.849 | 0.89 | 0.769 | 0.932 |
|  | ≥4 | 0.659 | 0.696 | 0.963 | 0.891 | 0.88 |
|  | ≥5 | 0.509 | 0.523 | 0.986 | 0.941 | 0.827 |
|  | ≥6 | 0.409 | 0.416 | 0.993 | 0.962 | 0.797 |

Table S3.2 – Test results of phq2&4 instrument for all probability thresholds on the primary dataset (PROACTIVE).

|  |  | PROACTIVE Test | | | | |
| --- | --- | --- | --- | --- | --- | --- |
| Pairing | Threshold | Youden | Sens | Spec | PPV | NPV |
| phq2&4 | ≥0.015 | 0 | 1 | 0 | 0.302 | 0 |
|  | ≥0.045 | 0.516 | 0.992 | 0.524 | 0.474 | 0.993 |
|  | ≥0.054 | 0.658 | 0.989 | 0.669 | 0.564 | 0.993 |
|  | ≥0.131 | 0.706 | 0.984 | 0.722 | 0.605 | 0.99 |
|  | ≥0.152 | 0.72 | 0.964 | 0.756 | 0.631 | 0.98 |
|  | ≥0.176 | 0.738 | 0.921 | 0.817 | 0.686 | 0.96 |
|  | ≥0.322 | 0.749 | 0.915 | 0.834 | 0.705 | 0.958 |
|  | ≥0.362 | 0.779 | 0.863 | 0.916 | 0.816 | 0.939 |
|  | ≥0.404 | 0.746 | 0.816 | 0.93 | 0.835 | 0.921 |
|  | ≥0.447 | 0.737 | 0.8 | 0.937 | 0.846 | 0.915 |
|  | ≥0.642 | 0.715 | 0.762 | 0.954 | 0.877 | 0.902 |
|  | ≥0.682 | 0.645 | 0.677 | 0.968 | 0.901 | 0.874 |
|  | ≥0.719 | 0.601 | 0.627 | 0.974 | 0.912 | 0.858 |
|  | ≥0.871 | 0.554 | 0.57 | 0.985 | 0.941 | 0.841 |
|  | ≥0.89 | 0.47 | 0.479 | 0.991 | 0.956 | 0.815 |
|  | ≥0.962 | 0.405 | 0.411 | 0.994 | 0.968 | 0.796 |

Table S3.3 – Test results of phq2&8 instrument for all probability thresholds on the primary dataset (PROACTIVE).

|  |  | PROACTIVE Test | | | | |
| --- | --- | --- | --- | --- | --- | --- |
| Pairing | Threshold | Youden | Sens | Spec | PPV | NPV |
| phq2&8 | ≥0.129 | 0 | 1 | 0 | 0.302 | 0 |
|  | ≥0.19 | 0.66 | 0.978 | 0.682 | 0.571 | 0.986 |
|  | ≥0.217 | 0.689 | 0.964 | 0.725 | 0.603 | 0.979 |
|  | ≥0.272 | 0.733 | 0.918 | 0.815 | 0.682 | 0.958 |
|  | ≥0.306 | 0.735 | 0.907 | 0.828 | 0.695 | 0.954 |
|  | ≥0.342 | 0.735 | 0.863 | 0.872 | 0.745 | 0.936 |
|  | ≥0.371 | 0.748 | 0.844 | 0.904 | 0.792 | 0.93 |
|  | ≥0.411 | 0.758 | 0.808 | 0.95 | 0.875 | 0.92 |
|  | ≥0.452 | 0.721 | 0.77 | 0.951 | 0.873 | 0.905 |
|  | ≥0.493 | 0.688 | 0.734 | 0.954 | 0.873 | 0.892 |
|  | ≥0.525 | 0.552 | 0.57 | 0.982 | 0.933 | 0.841 |
|  | ≥0.566 | 0.508 | 0.518 | 0.991 | 0.959 | 0.826 |
|  | ≥0.607 | 0.446 | 0.455 | 0.992 | 0.96 | 0.808 |
|  | ≥0.674 | 0.365 | 0.37 | 0.995 | 0.971 | 0.785 |
|  | ≥0.71 | 0.321 | 0.326 | 0.995 | 0.967 | 0.773 |
|  | ≥0.795 | 0.261 | 0.263 | 0.998 | 0.98 | 0.758 |

Table S4.1 – Generalisation results of PHQ-2 instrument for all discrete cutpoints on the PNS2013 dataset.

|  |  | PNS2013 | | | | |
| --- | --- | --- | --- | --- | --- | --- |
| Pairing | Cutpoint | Youden | Sens | Spec | PPV | NPV |
| PHQ-2 | ≥0 | 0 | 1 | 0 | 0.084 | 0 |
|  | ≥1 | 0.716 | 0.979 | 0.737 | 0.254 | 0.997 |
|  | ≥2 | 0.813 | 0.927 | 0.886 | 0.428 | 0.993 |
|  | ≥3 | 0.749 | 0.784 | 0.965 | 0.674 | 0.98 |
|  | ≥4 | 0.545 | 0.554 | 0.991 | 0.854 | 0.96 |
|  | ≥5 | 0.312 | 0.313 | 0.998 | 0.945 | 0.941 |
|  | ≥6 | 0.206 | 0.207 | 0.999 | 0.958 | 0.932 |

Table S4.2 – Generalisation results of phq2&4 instrument for all probability thresholds on the PNS2013 dataset.

|  |  | PNS2013 | | | | |
| --- | --- | --- | --- | --- | --- | --- |
| Pairing | Threshold | Youden | Sens | Spec | PPV | NPV |
| phq2&4 | ≥0.015 | 0 | 1 | 0 | 0.084 | 0 |
|  | ≥0.045 | 0.656 | 0.99 | 0.666 | 0.213 | 0.999 |
|  | ≥0.054 | 0.761 | 0.972 | 0.789 | 0.296 | 0.997 |
|  | ≥0.131 | 0.806 | 0.957 | 0.849 | 0.368 | 0.995 |
|  | ≥0.152 | 0.809 | 0.935 | 0.874 | 0.405 | 0.993 |
|  | ≥0.176 | 0.809 | 0.878 | 0.931 | 0.537 | 0.988 |
|  | ≥0.322 | 0.8 | 0.859 | 0.941 | 0.572 | 0.986 |
|  | ≥0.362 | 0.742 | 0.78 | 0.963 | 0.656 | 0.979 |
|  | ≥0.404 | 0.692 | 0.719 | 0.972 | 0.705 | 0.974 |
|  | ≥0.447 | 0.643 | 0.664 | 0.979 | 0.747 | 0.97 |
|  | ≥0.642 | 0.602 | 0.617 | 0.985 | 0.794 | 0.966 |
|  | ≥0.682 | 0.503 | 0.511 | 0.992 | 0.854 | 0.957 |
|  | ≥0.719 | 0.403 | 0.408 | 0.995 | 0.887 | 0.948 |
|  | ≥0.871 | 0.348 | 0.351 | 0.998 | 0.93 | 0.944 |
|  | ≥0.89 | 0.274 | 0.275 | 0.999 | 0.946 | 0.938 |
|  | ≥0.962 | 0.222 | 0.223 | 0.999 | 0.958 | 0.933 |

Table S4.3 – Generalisation results of phq2&8 instrument for all probability thresholds on the PNS2013 dataset.

|  |  | PNS2013 | | | | |
| --- | --- | --- | --- | --- | --- | --- |
| Pairing | Threshold | Youden | Sens | Spec | PPV | NPV |
| phq2&8 | ≥0.129 | 0 | 1 | 0 | 0.084 | 0 |
|  | ≥0.19 | 0.742 | 0.956 | 0.786 | 0.29 | 0.995 |
|  | ≥0.217 | 0.753 | 0.93 | 0.823 | 0.325 | 0.992 |
|  | ≥0.272 | 0.781 | 0.858 | 0.923 | 0.505 | 0.986 |
|  | ≥0.306 | 0.769 | 0.84 | 0.93 | 0.522 | 0.984 |
|  | ≥0.342 | 0.715 | 0.756 | 0.959 | 0.627 | 0.977 |
|  | ≥0.371 | 0.653 | 0.678 | 0.975 | 0.716 | 0.971 |
|  | ≥0.411 | 0.618 | 0.637 | 0.981 | 0.752 | 0.967 |
|  | ≥0.452 | 0.578 | 0.594 | 0.984 | 0.772 | 0.964 |
|  | ≥0.493 | 0.518 | 0.531 | 0.987 | 0.792 | 0.958 |
|  | ≥0.525 | 0.404 | 0.408 | 0.995 | 0.89 | 0.948 |
|  | ≥0.566 | 0.364 | 0.368 | 0.997 | 0.911 | 0.945 |
|  | ≥0.607 | 0.285 | 0.286 | 0.998 | 0.932 | 0.939 |
|  | ≥0.674 | 0.222 | 0.223 | 0.999 | 0.955 | 0.933 |
|  | ≥0.71 | 0.192 | 0.192 | 0.999 | 0.964 | 0.931 |
|  | ≥0.795 | 0.149 | 0.149 | 0.999 | 0.964 | 0.928 |

Table S5.1 – Generalisation results of PHQ-2 instrument for all discrete cutpoints on the PNS2019 dataset.

|  |  | PNS2019 | | | | |
| --- | --- | --- | --- | --- | --- | --- |
| Pairing | Cutpoint | Youden | Sens | Spec | PPV | NPV |
| PHQ-2 | ≥0 | 0 | 1 | 0 | 0.105 | 0 |
|  | ≥1 | 0.676 | 0.985 | 0.691 | 0.271 | 0.997 |
|  | ≥2 | 0.808 | 0.942 | 0.866 | 0.45 | 0.992 |
|  | ≥3 | 0.756 | 0.793 | 0.963 | 0.716 | 0.976 |
|  | ≥4 | 0.561 | 0.569 | 0.991 | 0.882 | 0.952 |
|  | ≥5 | 0.318 | 0.319 | 0.998 | 0.961 | 0.926 |
|  | ≥6 | 0.215 | 0.215 | 0.999 | 0.976 | 0.916 |

Table S5.2 – Generalisation results of phq2&4 instrument for all probability thresholds on the PNS2019 dataset.

|  |  | PNS2019 | | | | |
| --- | --- | --- | --- | --- | --- | --- |
| Pairing | Threshold | Youden | Sens | Spec | PPV | NPV |
| phq2&4 | ≥0.015 | 0 | 1 | 0 | 0.105 | 0 |
|  | ≥0.045 | 0.602 | 0.995 | 0.606 | 0.228 | 0.999 |
|  | ≥0.054 | 0.739 | 0.986 | 0.753 | 0.318 | 0.998 |
|  | ≥0.131 | 0.796 | 0.973 | 0.823 | 0.391 | 0.996 |
|  | ≥0.152 | 0.803 | 0.953 | 0.85 | 0.425 | 0.994 |
|  | ≥0.176 | 0.816 | 0.89 | 0.926 | 0.584 | 0.986 |
|  | ≥0.322 | 0.808 | 0.872 | 0.937 | 0.616 | 0.984 |
|  | ≥0.362 | 0.765 | 0.806 | 0.959 | 0.698 | 0.977 |
|  | ≥0.404 | 0.713 | 0.741 | 0.972 | 0.754 | 0.97 |
|  | ≥0.447 | 0.666 | 0.686 | 0.98 | 0.802 | 0.964 |
|  | ≥0.642 | 0.633 | 0.648 | 0.985 | 0.835 | 0.96 |
|  | ≥0.682 | 0.521 | 0.529 | 0.992 | 0.886 | 0.948 |
|  | ≥0.719 | 0.418 | 0.423 | 0.995 | 0.91 | 0.937 |
|  | ≥0.871 | 0.362 | 0.364 | 0.998 | 0.948 | 0.931 |
|  | ≥0.89 | 0.288 | 0.289 | 0.999 | 0.96 | 0.923 |
|  | ≥0.962 | 0.236 | 0.236 | 0.999 | 0.973 | 0.918 |

Table S5.3 – Generalisation results of phq2&8 instrument for all probability thresholds on the PNS2019 dataset.

|  |  | PNS2019 | | | | |
| --- | --- | --- | --- | --- | --- | --- |
| Pairing | Threshold | Youden | Sens | Spec | PPV | NPV |
| phq2&8 | ≥0.129 | 0 | 1 | 0 | 0.105 | 0 |
|  | ≥0.19 | 0.707 | 0.967 | 0.741 | 0.303 | 0.995 |
|  | ≥0.217 | 0.736 | 0.947 | 0.788 | 0.343 | 0.992 |
|  | ≥0.272 | 0.781 | 0.876 | 0.905 | 0.519 | 0.984 |
|  | ≥0.306 | 0.772 | 0.859 | 0.914 | 0.537 | 0.982 |
|  | ≥0.342 | 0.716 | 0.76 | 0.955 | 0.666 | 0.972 |
|  | ≥0.371 | 0.662 | 0.69 | 0.973 | 0.746 | 0.964 |
|  | ≥0.411 | 0.638 | 0.659 | 0.979 | 0.784 | 0.961 |
|  | ≥0.452 | 0.597 | 0.614 | 0.983 | 0.811 | 0.956 |
|  | ≥0.493 | 0.538 | 0.551 | 0.987 | 0.836 | 0.95 |
|  | ≥0.525 | 0.437 | 0.442 | 0.995 | 0.907 | 0.939 |
|  | ≥0.566 | 0.394 | 0.398 | 0.997 | 0.93 | 0.934 |
|  | ≥0.607 | 0.301 | 0.303 | 0.998 | 0.951 | 0.925 |
|  | ≥0.674 | 0.236 | 0.237 | 0.999 | 0.966 | 0.918 |
|  | ≥0.71 | 0.209 | 0.21 | 0.999 | 0.974 | 0.916 |
|  | ≥0.795 | 0.156 | 0.156 | 1 | 0.976 | 0.91 |

Table S6.1 – Generalisation results of PHQ-2 instrument for all discrete cutpoints on the Amazonas dataset.

|  |  | Amazonas | | | | |
| --- | --- | --- | --- | --- | --- | --- |
| Pairing | Cutpoint | Youden | Sens | Spec | PPV | NPV |
| PHQ-2 | ≥0 | 0 | 1 | 0 | 0.192 | 0 |
|  | ≥1 | 0.493 | 0.955 | 0.538 | 0.329 | 0.981 |
|  | ≥2 | 0.656 | 0.891 | 0.765 | 0.474 | 0.967 |
|  | ≥3 | 0.64 | 0.751 | 0.889 | 0.617 | 0.938 |
|  | ≥4 | 0.526 | 0.559 | 0.967 | 0.803 | 0.902 |
|  | ≥5 | 0.303 | 0.313 | 0.99 | 0.883 | 0.859 |
|  | ≥6 | 0.233 | 0.24 | 0.993 | 0.893 | 0.846 |

Table S6.2 – Generalisation results of phq2&4 instrument for all probability thresholds on the Amazonas dataset.

|  |  | Amazonas | | | | |
| --- | --- | --- | --- | --- | --- | --- |
| Pairing | Threshold | Youden | Sens | Spec | PPV | NPV |
| phq2&4 | ≥0.015 | 0 | 1 | 0 | 0.192 | 0 |
|  | ≥0.045 | 0.406 | 0.987 | 0.419 | 0.287 | 0.993 |
|  | ≥0.054 | 0.601 | 0.971 | 0.63 | 0.384 | 0.989 |
|  | ≥0.131 | 0.662 | 0.946 | 0.716 | 0.442 | 0.982 |
|  | ≥0.152 | 0.678 | 0.927 | 0.752 | 0.47 | 0.977 |
|  | ≥0.176 | 0.676 | 0.84 | 0.835 | 0.548 | 0.957 |
|  | ≥0.322 | 0.684 | 0.834 | 0.85 | 0.569 | 0.956 |
|  | ≥0.362 | 0.669 | 0.76 | 0.908 | 0.663 | 0.941 |
|  | ≥0.404 | 0.642 | 0.722 | 0.92 | 0.683 | 0.933 |
|  | ≥0.447 | 0.612 | 0.681 | 0.932 | 0.703 | 0.925 |
|  | ≥0.642 | 0.592 | 0.642 | 0.95 | 0.753 | 0.918 |
|  | ≥0.682 | 0.437 | 0.466 | 0.97 | 0.789 | 0.885 |
|  | ≥0.719 | 0.399 | 0.422 | 0.977 | 0.815 | 0.877 |
|  | ≥0.871 | 0.331 | 0.345 | 0.986 | 0.857 | 0.864 |
|  | ≥0.89 | 0.276 | 0.284 | 0.992 | 0.89 | 0.854 |
|  | ≥0.962 | 0.204 | 0.211 | 0.993 | 0.88 | 0.841 |

Table S6.3 – Generalisation results of phq2&8 instrument for all probability thresholds on the Amazonas dataset.

|  |  | Amazonas | | | | |
| --- | --- | --- | --- | --- | --- | --- |
| Pairing | Threshold | Youden | Sens | Spec | PPV | NPV |
| phq2&8 | ≥0.129 | 0 | 1 | 0 | 0.192 | 0 |
|  | ≥0.19 | 0.602 | 0.968 | 0.634 | 0.386 | 0.988 |
|  | ≥0.217 | 0.627 | 0.933 | 0.694 | 0.42 | 0.978 |
|  | ≥0.272 | 0.678 | 0.843 | 0.835 | 0.548 | 0.957 |
|  | ≥0.306 | 0.674 | 0.827 | 0.847 | 0.562 | 0.954 |
|  | ≥0.342 | 0.626 | 0.728 | 0.898 | 0.628 | 0.933 |
|  | ≥0.371 | 0.608 | 0.684 | 0.924 | 0.682 | 0.925 |
|  | ≥0.411 | 0.587 | 0.645 | 0.942 | 0.724 | 0.918 |
|  | ≥0.452 | 0.554 | 0.607 | 0.947 | 0.731 | 0.91 |
|  | ≥0.493 | 0.517 | 0.562 | 0.954 | 0.746 | 0.902 |
|  | ≥0.525 | 0.419 | 0.438 | 0.981 | 0.846 | 0.88 |
|  | ≥0.566 | 0.326 | 0.339 | 0.987 | 0.862 | 0.863 |
|  | ≥0.607 | 0.291 | 0.3 | 0.991 | 0.887 | 0.856 |
|  | ≥0.674 | 0.192 | 0.195 | 0.997 | 0.938 | 0.839 |
|  | ≥0.71 | 0.166 | 0.169 | 0.997 | 0.93 | 0.835 |
|  | ≥0.795 | 0.123 | 0.125 | 0.998 | 0.951 | 0.828 |

Table S7.1 – Generalisation results of PHQ-2 instrument for all discrete cutpoints on the Sao Paulo-Manaus dataset.

|  |  | Sao Paulo-Manaus | | | | |
| --- | --- | --- | --- | --- | --- | --- |
| Pairing | Cutpoint | Youden | Sens | Spec | PPV | NPV |
| PHQ-2 | ≥0 | 0 | 1 | 0 | 0.085 | 0 |
|  | ≥1 | 0.683 | 0.957 | 0.725 | 0.245 | 0.995 |
|  | ≥2 | 0.766 | 0.906 | 0.86 | 0.375 | 0.99 |
|  | ≥3 | 0.755 | 0.821 | 0.934 | 0.536 | 0.982 |
|  | ≥4 | 0.628 | 0.65 | 0.979 | 0.738 | 0.968 |
|  | ≥5 | 0.44 | 0.444 | 0.995 | 0.897 | 0.951 |
|  | ≥6 | 0.364 | 0.368 | 0.996 | 0.896 | 0.944 |

Table S7.2 – Generalisation results of phq2&4 instrument for all probability thresholds on the Sao Paulo-Manaus dataset.

|  |  | Sao Paulo-Manaus | | | | |
| --- | --- | --- | --- | --- | --- | --- |
| Pairing | Threshold | Youden | Sens | Spec | PPV | NPV |
| phq2&4 | ≥0.015 | 0 | 1 | 0 | 0.085 | 0 |
|  | ≥0.045 | 0.569 | 0.983 | 0.587 | 0.181 | 0.997 |
|  | ≥0.054 | 0.687 | 0.957 | 0.73 | 0.248 | 0.995 |
|  | ≥0.131 | 0.739 | 0.957 | 0.782 | 0.289 | 0.995 |
|  | ≥0.152 | 0.754 | 0.949 | 0.806 | 0.312 | 0.994 |
|  | ≥0.176 | 0.725 | 0.846 | 0.879 | 0.393 | 0.984 |
|  | ≥0.322 | 0.719 | 0.829 | 0.89 | 0.411 | 0.982 |
|  | ≥0.362 | 0.699 | 0.769 | 0.929 | 0.503 | 0.977 |
|  | ≥0.404 | 0.681 | 0.744 | 0.937 | 0.524 | 0.975 |
|  | ≥0.447 | 0.673 | 0.726 | 0.946 | 0.556 | 0.974 |
|  | ≥0.642 | 0.6 | 0.632 | 0.967 | 0.643 | 0.966 |
|  | ≥0.682 | 0.536 | 0.556 | 0.98 | 0.722 | 0.96 |
|  | ≥0.719 | 0.491 | 0.504 | 0.987 | 0.776 | 0.955 |
|  | ≥0.871 | 0.436 | 0.444 | 0.991 | 0.825 | 0.951 |
|  | ≥0.89 | 0.378 | 0.385 | 0.994 | 0.849 | 0.946 |
|  | ≥0.962 | 0.338 | 0.342 | 0.996 | 0.889 | 0.942 |

Table S7.3 – Generalisation results of phq2&8 instrument for all probability thresholds on the Sao Paulo-Manaus dataset.

|  |  | Sao Paulo-Manaus | | | | |
| --- | --- | --- | --- | --- | --- | --- |
| Pairing | Threshold | Youden | Sens | Spec | PPV | NPV |
| phq2&8 | ≥0.129 | 0 | 1 | 0 | 0.085 | 0 |
|  | ≥0.19 | 0.703 | 0.957 | 0.746 | 0.259 | 0.995 |
|  | ≥0.217 | 0.725 | 0.949 | 0.776 | 0.282 | 0.994 |
|  | ≥0.272 | 0.787 | 0.906 | 0.881 | 0.414 | 0.99 |
|  | ≥0.306 | 0.776 | 0.889 | 0.887 | 0.423 | 0.989 |
|  | ≥0.342 | 0.757 | 0.838 | 0.919 | 0.49 | 0.984 |
|  | ≥0.371 | 0.761 | 0.821 | 0.94 | 0.561 | 0.983 |
|  | ≥0.411 | 0.729 | 0.778 | 0.952 | 0.599 | 0.979 |
|  | ≥0.452 | 0.717 | 0.761 | 0.956 | 0.618 | 0.977 |
|  | ≥0.493 | 0.679 | 0.718 | 0.961 | 0.632 | 0.973 |
|  | ≥0.525 | 0.477 | 0.487 | 0.99 | 0.814 | 0.954 |
|  | ≥0.566 | 0.387 | 0.393 | 0.994 | 0.852 | 0.946 |
|  | ≥0.607 | 0.329 | 0.333 | 0.996 | 0.886 | 0.941 |
|  | ≥0.674 | 0.204 | 0.205 | 0.999 | 0.96 | 0.931 |
|  | ≥0.71 | 0.179 | 0.179 | 0.999 | 0.955 | 0.929 |
|  | ≥0.795 | 0.145 | 0.145 | 0.999 | 0.944 | 0.926 |

Table S8.1 – Generalisation results of PHQ-2 instrument for all discrete cutpoints on the Mexican Medical Students dataset.

|  |  | MexMedStudents | | | | |
| --- | --- | --- | --- | --- | --- | --- |
| Pairing | Cutpoint | Youden | Sens | Spec | PPV | NPV |
| PHQ-2 | ≥0 | 0 | 1 | 0 | 0.248 | 0 |
|  | ≥1 | 0.199 | 0.99 | 0.21 | 0.292 | 0.984 |
|  | ≥2 | 0.463 | 0.906 | 0.557 | 0.403 | 0.947 |
|  | ≥3 | 0.492 | 0.557 | 0.935 | 0.738 | 0.865 |
|  | ≥4 | 0.243 | 0.255 | 0.988 | 0.875 | 0.801 |
|  | ≥5 | 0.094 | 0.094 | 1 | 1 | 0.77 |
|  | ≥6 | 0.031 | 0.031 | 1 | 1 | 0.758 |

Table S8.2 – Generalisation results of phq2&4 instrument for all probability thresholds on the Mexican Medical Students dataset.

|  |  | MexMedStudents | | | | |
| --- | --- | --- | --- | --- | --- | --- |
| Pairing | Threshold | Youden | Sens | Spec | PPV | NPV |
| phq2&4 | ≥0.015 | 0 | 1 | 0 | 0.248 | 0 |
|  | ≥0.045 | 0.088 | 0.995 | 0.093 | 0.266 | 0.982 |
|  | ≥0.054 | 0.392 | 0.979 | 0.412 | 0.355 | 0.984 |
|  | ≥0.131 | 0.429 | 0.974 | 0.455 | 0.371 | 0.981 |
|  | ≥0.152 | 0.448 | 0.927 | 0.521 | 0.389 | 0.956 |
|  | ≥0.176 | 0.619 | 0.755 | 0.864 | 0.647 | 0.915 |
|  | ≥0.322 | 0.623 | 0.755 | 0.868 | 0.653 | 0.915 |
|  | ≥0.362 | 0.612 | 0.734 | 0.878 | 0.665 | 0.909 |
|  | ≥0.404 | 0.481 | 0.516 | 0.966 | 0.832 | 0.858 |
|  | ≥0.447 | 0.481 | 0.516 | 0.966 | 0.832 | 0.858 |
|  | ≥0.642 | 0.429 | 0.453 | 0.976 | 0.861 | 0.844 |
|  | ≥0.682 | 0.313 | 0.318 | 0.995 | 0.953 | 0.815 |
|  | ≥0.719 | 0.205 | 0.208 | 0.997 | 0.952 | 0.792 |
|  | ≥0.871 | 0.179 | 0.182 | 0.997 | 0.946 | 0.787 |
|  | ≥0.89 | 0.097 | 0.099 | 0.998 | 0.95 | 0.771 |
|  | ≥0.962 | 0.068 | 0.068 | 1 | 1 | 0.765 |

Table S8.3 – Generalisation results of phq2&8 instrument for all probability thresholds on the Mexican Medical Students dataset.

|  |  | MexMedStudents | | | | |
| --- | --- | --- | --- | --- | --- | --- |
| Pairing | Threshold | Youden | Sens | Spec | PPV | NPV |
| phq2&8 | ≥0.129 | 0 | 1 | 0 | 0.248 | 0 |
|  | ≥0.19 | 0.367 | 0.969 | 0.399 | 0.347 | 0.975 |
|  | ≥0.217 | 0.413 | 0.938 | 0.476 | 0.371 | 0.958 |
|  | ≥0.272 | 0.659 | 0.802 | 0.857 | 0.65 | 0.929 |
|  | ≥0.306 | 0.649 | 0.781 | 0.868 | 0.661 | 0.923 |
|  | ≥0.342 | 0.499 | 0.536 | 0.962 | 0.824 | 0.863 |
|  | ≥0.371 | 0.448 | 0.474 | 0.974 | 0.858 | 0.849 |
|  | ≥0.411 | 0.445 | 0.469 | 0.976 | 0.865 | 0.848 |
|  | ≥0.452 | 0.347 | 0.354 | 0.993 | 0.944 | 0.823 |
|  | ≥0.493 | 0.243 | 0.245 | 0.998 | 0.979 | 0.8 |
|  | ≥0.525 | 0.219 | 0.219 | 1 | 1 | 0.795 |
|  | ≥0.566 | 0.182 | 0.182 | 1 | 1 | 0.788 |
|  | ≥0.607 | 0.13 | 0.13 | 1 | 1 | 0.777 |
|  | ≥0.674 | 0.089 | 0.089 | 1 | 1 | 0.769 |
|  | ≥0.71 | 0.057 | 0.057 | 1 | 1 | 0.763 |
|  | ≥0.795 | 0.026 | 0.026 | 1 | 1 | 0.757 |

Table S9.1 – Generalisation results of PHQ-2 instrument for all discrete cutpointss on the Jockey Club JoyAge dataset.

|  |  | JC JoyAge | | | | |
| --- | --- | --- | --- | --- | --- | --- |
| Pairing | Cutpoint | Youden | Sens | Spec | PPV | NPV |
| PHQ-2 | ≥0 | 0 | 1 | 0 | 0.192 | 0 |
|  | ≥1 | 0.258 | 0.99 | 0.268 | 0.243 | 0.991 |
|  | ≥2 | 0.452 | 0.958 | 0.494 | 0.31 | 0.98 |
|  | ≥3 | 0.595 | 0.822 | 0.773 | 0.462 | 0.948 |
|  | ≥4 | 0.519 | 0.613 | 0.906 | 0.606 | 0.908 |
|  | ≥5 | 0.296 | 0.313 | 0.983 | 0.814 | 0.858 |
|  | ≥6 | 0.168 | 0.173 | 0.995 | 0.897 | 0.835 |

Table S9.2 – Generalisation results of phq2&4 instrument for all probability thresholds on the Jockey Club JoyAge dataset.

|  |  | JC JoyAge | | | | |
| --- | --- | --- | --- | --- | --- | --- |
| Pairing | Threshold | Youden | Sens | Spec | PPV | NPV |
| phq2&4 | ≥0.015 | 0 | 1 | 0 | 0.192 | 0 |
|  | ≥0.045 | 0.149 | 0.999 | 0.15 | 0.218 | 0.998 |
|  | ≥0.054 | 0.265 | 0.994 | 0.271 | 0.244 | 0.995 |
|  | ≥0.131 | 0.34 | 0.989 | 0.351 | 0.265 | 0.993 |
|  | ≥0.152 | 0.396 | 0.984 | 0.412 | 0.284 | 0.991 |
|  | ≥0.176 | 0.579 | 0.933 | 0.646 | 0.384 | 0.976 |
|  | ≥0.322 | 0.59 | 0.915 | 0.675 | 0.4 | 0.971 |
|  | ≥0.362 | 0.593 | 0.889 | 0.704 | 0.416 | 0.964 |
|  | ≥0.404 | 0.585 | 0.792 | 0.793 | 0.476 | 0.942 |
|  | ≥0.447 | 0.581 | 0.695 | 0.886 | 0.591 | 0.924 |
|  | ≥0.642 | 0.572 | 0.671 | 0.9 | 0.615 | 0.92 |
|  | ≥0.682 | 0.508 | 0.585 | 0.923 | 0.643 | 0.904 |
|  | ≥0.719 | 0.334 | 0.366 | 0.968 | 0.731 | 0.866 |
|  | ≥0.871 | 0.319 | 0.331 | 0.987 | 0.862 | 0.862 |
|  | ≥0.89 | 0.236 | 0.244 | 0.992 | 0.879 | 0.847 |
|  | ≥0.962 | 0.159 | 0.162 | 0.997 | 0.936 | 0.834 |

Table S9.3 – Generalisation results of phq2&8 instrument for all probability thresholds on the Jockey Club JoyAge dataset.

|  |  | JC JoyAge | | | | |
| --- | --- | --- | --- | --- | --- | --- |
| Pairing | Threshold | Youden | Sens | Spec | PPV | NPV |
| phq2&8 | ≥0.129 | 0 | 1 | 0 | 0.192 | 0 |
|  | ≥0.19 | 0.306 | 0.978 | 0.328 | 0.257 | 0.984 |
|  | ≥0.217 | 0.329 | 0.973 | 0.357 | 0.264 | 0.982 |
|  | ≥0.272 | 0.537 | 0.847 | 0.69 | 0.393 | 0.95 |
|  | ≥0.306 | 0.535 | 0.841 | 0.695 | 0.395 | 0.948 |
|  | ≥0.342 | 0.553 | 0.774 | 0.779 | 0.454 | 0.936 |
|  | ≥0.371 | 0.489 | 0.561 | 0.928 | 0.649 | 0.899 |
|  | ≥0.411 | 0.486 | 0.557 | 0.928 | 0.649 | 0.898 |
|  | ≥0.452 | 0.462 | 0.528 | 0.934 | 0.655 | 0.893 |
|  | ≥0.493 | 0.329 | 0.375 | 0.954 | 0.66 | 0.865 |
|  | ≥0.525 | 0.188 | 0.197 | 0.992 | 0.85 | 0.839 |
|  | ≥0.566 | 0.173 | 0.18 | 0.992 | 0.849 | 0.836 |
|  | ≥0.607 | 0.133 | 0.137 | 0.996 | 0.881 | 0.83 |
|  | ≥0.674 | 0.071 | 0.072 | 0.999 | 0.951 | 0.819 |
|  | ≥0.71 | 0.058 | 0.058 | 0.999 | 0.959 | 0.817 |
|  | ≥0.795 | 0.031 | 0.031 | 1 | 1 | 0.813 |
