# Supplementary material for: Patient Health Questionnaire-9 Item Pairing Predictiveness for Prescreening Depressive Symptomatology: Machine Learning Analysis
Source: JMIR Ment Health. 2023 Oct 19;10:e48444. doi: 10.2196/48444 (PMC10623235; doi:10.2196/48444)
Supplement: Multimedia Appendix 3 [file mental_v10i1e48444_app3.doc]

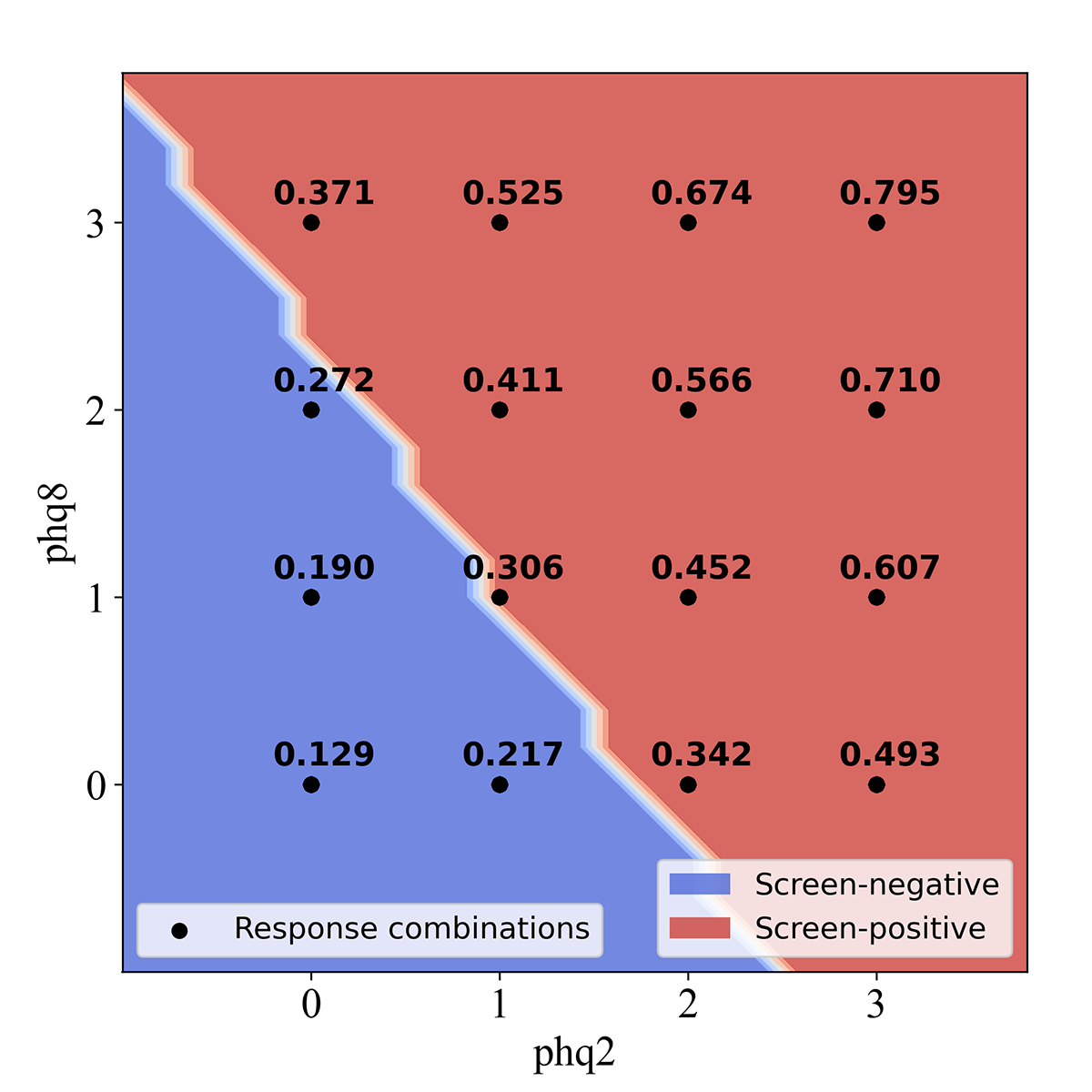

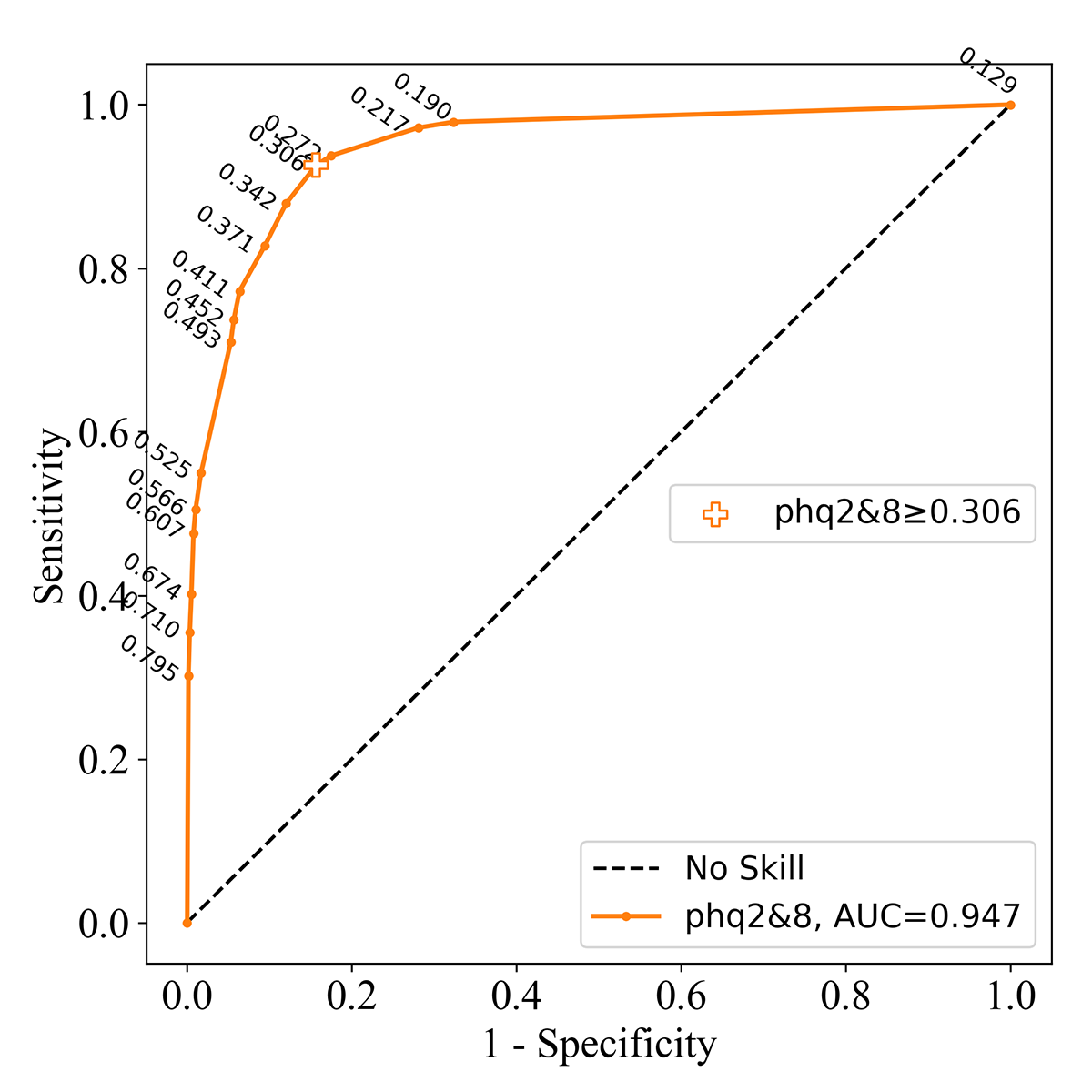
Figure S2 - Probability thresholds on the phq2&8 machine learning receiver operating characteristic curve on the primary datasets training set. Each threshold represents a decision boundary in the input feature space.

Figure S1 – Input feature space showing all 16 possible item response combinations for the phq2&8. phq2, Patient Health Questionnaire-9 item 2; phq8, Patient Health Questionnaire-9 item 8.
